# Chemodiversity, pharmacological activity, and biosynthesis of specialized metabolites from medicinal model fungi *Ganoderma lucidum*

**DOI:** 10.1186/s13020-024-00922-0

**Published:** 2024-03-22

**Authors:** Yupeng Du, Lixia Tian, Yu Wang, Zhenhao Li, Zhichao Xu

**Affiliations:** 1 Key Laboratory of Saline-Alkali Vegetation Ecology Restoration (Northeast Forestry University), Ministry of Education, Harbin, 150040 China; 2 College of Life Science, Northeast Forestry University, Harbin, 150040 China; 3 School of Pharmaceutical Sciences, Guizhou University, Guiyang, 550025 China; 4 Institute of Medicinal Plant Development, Chinese Academy of Medical Sciences & Peking Union Medical College, Beijing, 100193 China; 5 ShouXianGu Botanical Drug Institute, Hangzhou, 311100 China

**Keywords:** *Ganoderma lucidum*, Ganoderic acids, Chemodiversity, Pharmacological activity, Biosynthesis

## Abstract

*Ganoderma lucidum* is a precious fungus, particularly valued for its dual use as both medicine and food. Ganoderic acids (GAs), the distinctive triterpenoids found in the *Ganoderma* genus, exhibit a wide range of pharmacological activities. However, the limited resources of GAs restrict their clinic usage and drug discovery. In this review, we presented a comprehensive summary focusing on the diverse structures and pharmacological activity of GAs in *G. lucidum*. Additionally, we discussed the latest advancements in the elucidation of GA biosynthesis, as well as the progress in heterosynthesis and liquid fermentation methods aimed at further increasing GA production. Furthermore, we summarized the omics data, genetic transformation system, and cultivation techniques of *G. lucidum*, described as medicinal model fungi. The understanding of Ganoderic acids chemodiversity and biosynthesis in medicinal model fungi *Ganoderma lucidum* will provide important insights into the exploration and utilization of natural products in medicinal fungi.

**Supplementary Information:**

The online version contains supplementary material available at 10.1186/s13020-024-00922-0.

## Introduction

*Ganoderma lucidum*, a medicinal mushroom, belongs to the family Ganodermataceae of the Basidiomycota. The name *Ganoderma* originates from Greek, meaning shiny, glowing skin (“gános”—shiny, glowing, brightness; and “dérma”—skin). While there are ongoing debates about the classification of the *Ganoderma* genus and the naming of different *Ganoderma* species [1], we follows the designation of *G. lucidum* in accordance with the Chinese Pharmacopoeia. In nature, it mostly grows on living and dead wood of deciduous species under high humidity and indistinct lighting [2]. The fruiting bodies of *G. lucidum* have been used in Traditional Chinese Medicine for over 2000 years. And, *G. lucidum* contains an abundance of natural bioactive components, mainly polysaccharides, terpenoids, glycopeptides, nucleotides, steroids, and unsaturated fatty acids, as well as small amounts of amino acids and proteins [3]. *G. lucidum* produces a group of highly oxygenated lanostane-type triterpenoids, including triterpenoid acids, alcohols, and aldehydes, as well as their derivatives. These oxygenated lanostane-type triterpenoids (e. g. ganoderic acids, GAs) are major bioactive compounds in *G. lucidum*, which have been shown to exhibit cytotoxic [4], cytostatic [3], antiviral [5], antibacterial [6], anticancer [7], and hypoglycemic effects [8]. The huge market demand for *G. lucidum* is increasing due to its excellent medical values, and the annual sale of products derived from *G. lucidum* was estimated to be more than 2.5 billion U.S. dollars [9, 10].

GAs are well recognized as a main group of unique bioactive compounds in *Ganoderma*, and several individual GAs have been reported to possess important biological and pharmacological activities, including antitumor, anti-metastasis, and anti-HIV effects in both in vitro and in vivo [11]. Despite the outstanding medicinal value of GAs, their low production has become a bottleneck for their widespread application. GAs are traditionally extracted from the fruiting bodies and spores of their native producing host, *G. lucidum*, but the field cultivation of the mushroom is labor-intensive and time-consuming while the product quality is very difficult to guarantee. Recently, metabolic engineering of GAs and molecular breeding of new *G. lucidum* variety with high-yield GAs have been promising approaches for GA production. However, the complete biosynthetic pathway of GAs has been an unresolved puzzling question for nearly 30 years since then, which might include a series of oxidation modifications of lanosterol [12]. To date, only a few genes involved in the biotransformation of lanosterol to GAs have been characterized, and the further biosynthetic steps remain to be extremely challenging. Due to the elucidation of genome data, transgenic system, and specifically medicinal triterpenoids, *G. lucidum* has been described as the ideal model for studying the molecular mechanism of medicinal fungi [13]. Here, we systematically summarize the chemical structures, diversity, and pharmacological activities of GAs, and review the biosynthetic progress and metabolic engineering of GAs in *G. lucidum*. This review aims to enhance understanding of GA diversity and biosynthesis, providing a basis for further development and utilization of GAs.

## Chemical structures and diversity of lanostane-type triterpenoids from *G. lucidum*

**Fig. 1:**
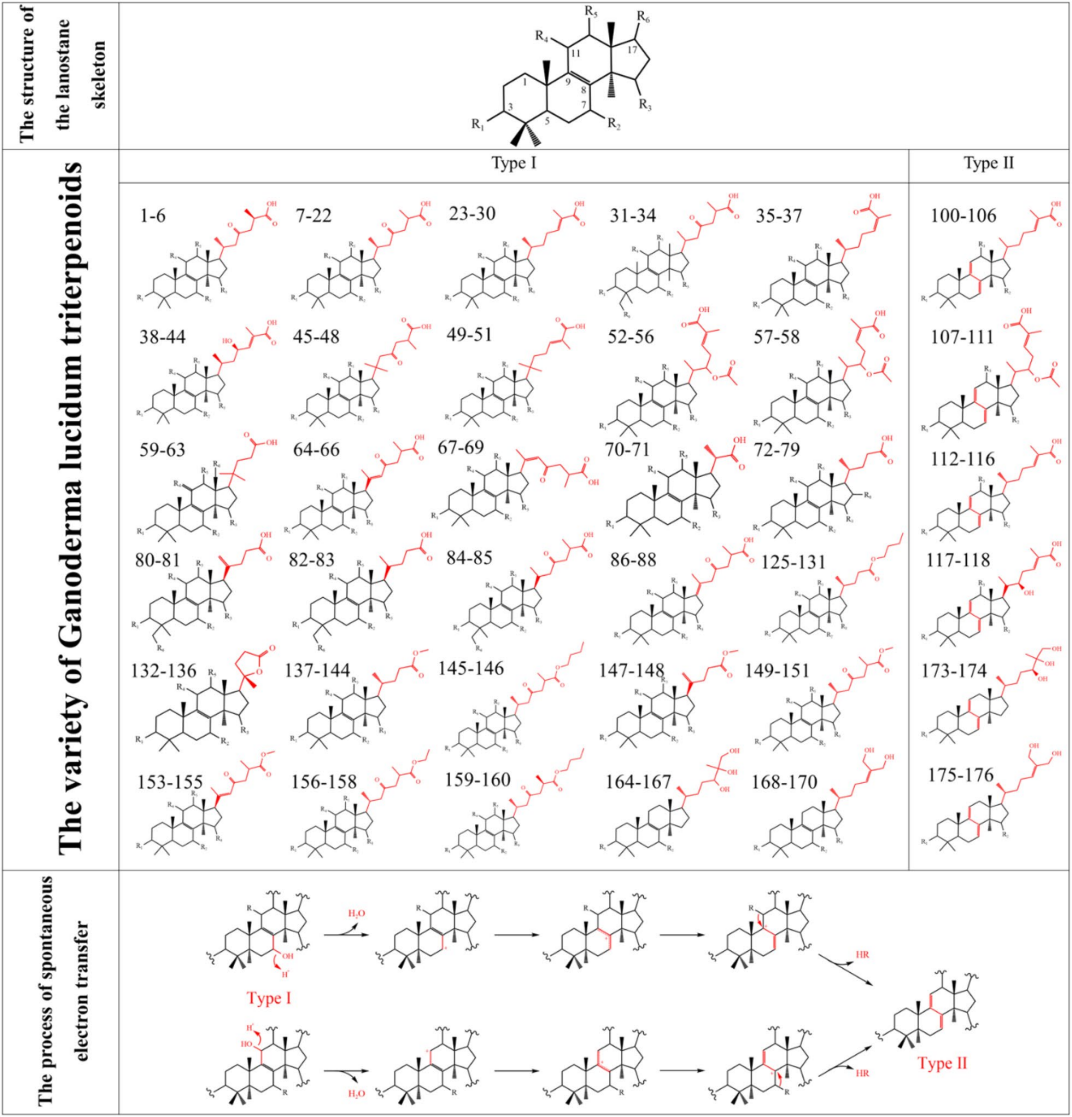
Chemical structures and diversity of active compounds from *G. lucidum* and possible conversion methods from Type I to Type II. The compounds correspond to their respective serial numbers in Additional file 1: Fig. S1 and Table S1. The spontaneous electron transfer process of type I GAs to type II GAs was presented

In 1982, Takashi Kubota successfully isolated two novel lanostane-type triterpenes [14], namely ganoderic acid A and B (1,2), from the epidermis of *G. lucidum*, marking the initial discovery of these compounds in that particular fungal species. Subsequently, a substantial number of approximately 200 lanostane-type triterpenoid compounds (Fig. 1) have been identified and isolated from various parts of *G. lucidum*, including the fruiting bodies, mycelia, and spores. Among these triterpenes, those containing the carboxyl group are known as Ganoderic acid (GA), while the rest are derivatives. These ganoderic acids feature lanostane skeletons with trans configurations of rings A/B, B/C, and C/D, alongside 10β-CH3, 13β-CH3, 14α-CH3, and 17β substitutions. GAs consist of a lanosterol skeleton with diverse postmodifications, such as hydroxylation, methylation, and acetylation. Based on these modifications, GAs are categorized into two types. Type I GAs (1–99, 125–177, 187–196) possess only one double bond on their tetracyclic rings, whereas type II GAs (100–124, 173–181) exhibit conjugated double bonds on their tetracyclic rings [15]. Compared with type I GAs, type II GAs harbor more sophisticated modifications and exhibit stronger anti-tumor activities in general [16]. Type II GAs undergo spontaneous formation from unstable intermediates with a hydroxyl group at either C-7 or C-11. The creation of conjugated double bonds (C7=C8, C9=C11) involves a cascade reaction of carbon cation generation, migration, and elimination. The hydroxyl groups (OH-7 or OH-11) are presumed to be protonated and converted into carbocation intermediates, which then migrate to C-8 or C-9, ultimately forming the corresponding carbocation intermediates and generating conjugated double bonds through elimination [17]. The diversity of GAs is particularly rooted in the myriad of distinct substitutions at C-17. Consequently, the functional groups present in C-17 substitutions allow for further categorization of GAs into compounds such as ganoderic acids, ganoderiol, ganoderal and ganolactone [12].

## Pharmacological activity of GAs

**Fig. 2:**
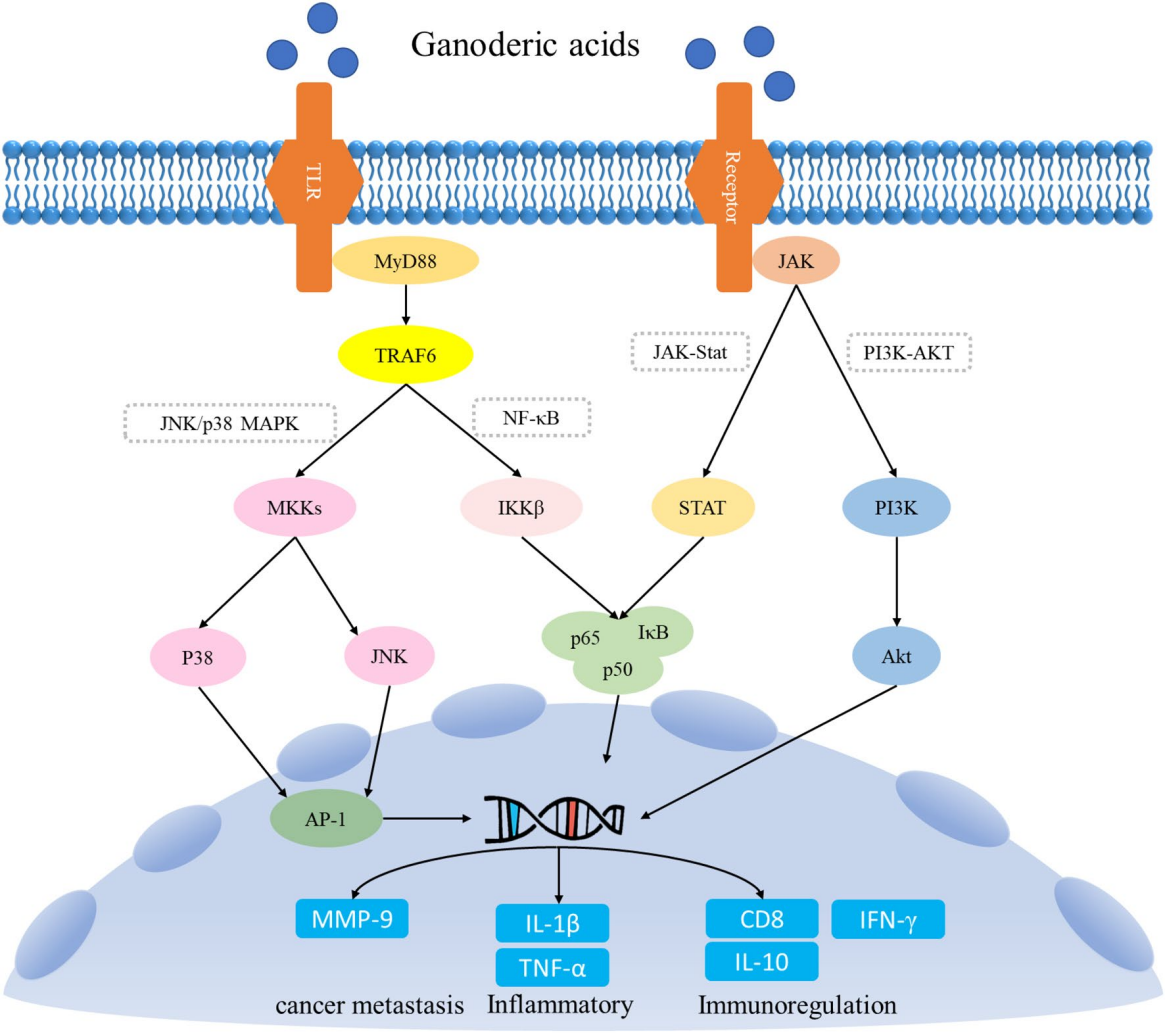
Signaling pathways and active substances involved in the main pharmacological activities of ganoderic acid. *TLR* toll-like receptors, *MyD88* myeloiddifferentiationfactor88, *JAK* janus kinase, *TRAF6* TNF receptor associated factor 6, *MAPK* mitogen-activated protein kinase, *NF-κB* nuclear factor kappa-B, *MKK* MAP kinase kinase, *IKKβ* inhibitor kappa B kinaseβ, *STAT* signal transducers and activators of transcription, *PI3K* phosphatidylinositide 3-kinases, *p38* p38 mitogen-activated protein kinase, *JNK* c-Jun N-terminal kinase, *p50* NF-κB1, *p65* RelA, *IκB* inhibitor of NF-κB, *Akt* protein kinase B, *AP-1* activator protein-1, *MMP-9* matrix metalloproteinase-9, *IL-1β* interleukin-1β, *TNF-α* tumor necrosis factor-α, *CD8* cluster of differentiation 8, *IL-10* interleukin 10, *IFN-γ* interferon γ

*Ganoderma lucidum* contains a wide range of active triterpenoid compounds, with various pharmacological properties, such as combating tumors [18], inhibiting microbial growth [19], regulating blood sugar levels [20], reducing lipid levels [21], alleviating inflammation [22], modulating the immune system [23], and safeguarding liver health [24]. The extensive bioactivity of these compounds makes *G. lucidum* a highly valuable and sought-after medicinal fungus with immense potential for therapeutic applications (Fig. 2).

Within this spectrum of compounds, GA-A has advanced to Phase I of development, indicating its progression toward clinical trials. Meanwhile, GA-C2 is currently undergoing preclinical evaluation, suggesting ongoing research before human trials. On the other hand, GA-D, GA-DM, GA-X, and GA-Y are all undergoing biological testing, reflecting early-stage assessment of their pharmacological activities and safety profiles. These distinct development stages reflect the concerted efforts to harness the therapeutic potential of *G. lucidum* compounds for various health benefits.

### Antitumor

Pharmacological and clinical investigations have confirmed that anticancer effects of GAs generally include inhibiting tumor cell proliferation, inducing tumor cell apoptosis, and preventing cancer cell metastasis [25]. *G. lucidum* extract has exhibited anti-proliferative effects by inducing DNA damage, G1 cell cycle arrest, and apoptosis in human breast cancer cells [26]. Additionally, GAs resulted in a prolonged G2 cell cycle phase and strong growth inhibition, indicating that GAs can slow down the cell cycle to prevent cancer cell proliferation [27]. GAs have been also found to exhibit antitumor effects by inducing apoptosis in tumor cells via regulating various pathways and factors involved in cell survival and apoptosis. MAPK and PI3K/Akt pathways [28–30] are crucial in regulating cell survival and apoptosis, and GAs could activate JNK and p38 MAPK pathways, while inhibiting the PI3K/Akt pathway, leading to apoptosis in tumor cells [31]. Additionally, GAs could regulate the expression of transcription factors such as p53, NF-κB, and AP-1 [32–34], which are involved in the regulation of tumor cell apoptosis and survival. Furthermore, GAs could regulate Bax and Bcl-2 expression, inhibiting *Bcl-2* and activating *Bax*, to trigger tumor cell apoptosis via mitochondrial channel opening and cytochrome C release [35]. Overall, *G. lucidum* extract and GAs exert antitumor effects by regulating multiple pathways and factors involved in tumor cell survival and apoptosis. GA-T, as an important component of GAs, has been found to have a wide range of anticancer effects. GA-T has demonstrated effectiveness in promoting apoptosis in lung cancer cells through mitochondria-mediated mechanisms [36]. Additionally, GA-T has been shown to inhibit the expression of matrix metalloproteinases (MMP-9), which can promote cancer metastasis by degrading the extracellular matrix [37].

### Anti-inflammatory

Inflammation is a common pathological process in many diseases, including cardiovascular disease, diabetes, and cancer [38]. GAs can reduce inflammation by modulating multiple pathways in the body. The NF-κB signaling pathway is one of the most important pathways in the inflammatory response [39]. Studies indicate that GAs can inhibit the activation of NF-κB through various mechanisms, thereby suppressing the inflammatory response. For instance, interleukin-6 (IL-6) is a crucial pro-inflammatory cytokine that gets released during infections or tissue damage and triggers innate and acquired immune responses. Similarly, IL-1β also has strong pro-inflammatory activity and can induce several pro-inflammatory mediators like cytokines and chemokines. By regulating the expression of IKK and IκB, GA-A can prevent the activation of NF-κB and thereby suppress the production of cytokines like IL-6 and IL-1β [40]. GA-A can also inhibit NF-κB nuclear translocation, reducing inflammatory mediator production [41]. In addition to suppressing the production of pro-inflammatory cytokines, GA-A can directly inhibit the activity of NLRP3 inflammasomes [42]. GAs have been reported to exhibit numerous anti-inflammatory effects and can regulate different signaling pathways related to inflammation to produce its effects [43, 44].

### Immunoregulation

GAs primarily target various cytokines to produce immunomodulatory effects. For instance, GA-Me can trigger apoptosis in T cells, leading to a decrease in CD8+ T cell activation and an increase in Treg-mediated immunosuppression [45]. GA-A has also been found to enhance the cytotoxicity of T cells [46]. Moreover, GAs have been found to specifically regulate cytokines, unlike other compounds that have a general suppressive effect on cytokines. For instance, treatment with GA-β and dexamethasone had different effects on cytokines in asthma cells. While dexamethasone broadly inhibited cytokines, GA-C1 increased the production of immune regulation-associated cytokines such as IL-10 and IFN-γ, and reduced the production of IL-5, which is associated with asthma attacks [47].

### Other pharmacological activities

GAs have also exhibited a diverse range of pharmacological effects such as reducing blood glucose levels [48], lowering lipid levels [49], and providing protection to the liver [50] and kidneys [51]. These findings highlight the multifunctional potential of GAs as a natural remedy and provide a strong scientific foundation for its varied applications. Lowering blood lipid and anti-obesity: GA-A inhibits the expression of sterol regulatory element-binding proteins (SREBPs) and reduces cellular levels of cholesterol and fatty acids in vitro, potentially aiding in lowering blood lipid and combating obesity [52]; GA-A also improves weight gain and reduces fat accumulation in the liver or adipose tissue, and improves lipid levels and insulin sensitivity in high-fat diet-induced obese mice [53]. Protecting the liver and kidney: GA-A protects against cyclophosphamide-induced hepatotoxicity and alcoholic liver injury by modulating lipid metabolism [15] and intestinal microbial composition [24]. In addition, GA-A can protect against bleomycin (BLM)-induced pulmonary fibrosis and hinder renal fibrosis by suppressing the TGF-β/Smad and MAPK signaling pathways, and can also delay the progression of polycystic kidney disease [51].

## GA biosynthesis

**Fig. 3:**
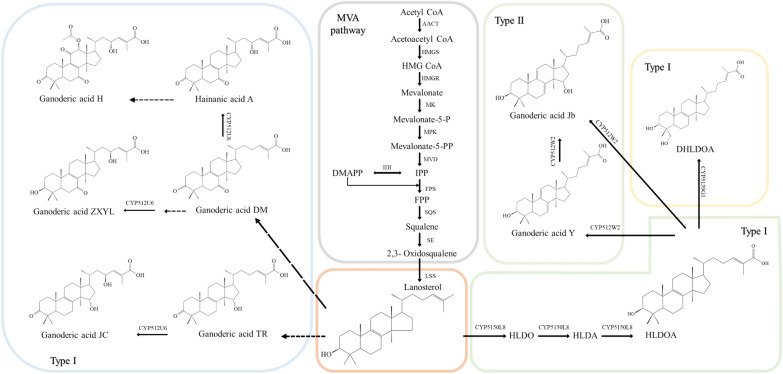
Proposed pathways for ganoderic acid biosynthesis. *AACT* actyl-CoA C-acetyltransferase, *HMGS* 3-hydroxy-3-methylglutaryl coenthase A synthase, *HMGR* 3-hydroxy-3-methyl glutaryl coenzyme A reductase, *MK* mevalonate kinase, *MPK* phosphomevalonate kinase, *MVD* mevalonate pyrophosphate decarboxylase, *IPP* isopentenyl diphosphate, *IDI* diphosphateisomerase, *DMAPP* dimethylallyl pyrophosphate, *FPS* farnesyl pyrophosphate synthase, *FPP* farnesyl pyrophosphate, *SQS* squalene synthase, *SE* squalene epoxidase, *LSS* lanosterol synthase

The biosynthesis of GAs involves the upstream mevalonate (MVA) pathway for the production of lanosterol, which is then converted into chemically diverse GAs by various oxidized modifications [13]. The enzymes and genes involved in the upstream process are identified, but the downstream biotransformation process remains largely unknown (Fig. 3).

13C isotope labeling revealed that GA biosynthesis starts from the MVA pathway [54]. The process of synthesizing lanosterol from acetyl-CoA involves several steps. First, three molecules of acetyl-CoA undergo two condensations to form 3-hydroxy-3-methylglutaryl-CoA (HMG-CoA). HMG-CoA is then converted to mevalonic acid (MVA) by the action of HMG-CoA reductase (HMGR). MVA is then phosphorylated by mevalonate kinase (MK) and hydroxymethylglutaryl-CoA reductase (MPK), and decarboxylated by mevalonate pyrophosphate decarboxylase (MVD) to form isopentenyl pyrophosphate (IPP). IPP can be converted to dimethylallyl pyrophosphate (DMAPP) by IPP isomerase (IDI). Farnesyl pyrophosphate (FPP) is formed by the head-to-tail condensation of two molecules of IPP and one molecule of DMAPP, catalyzed by farnesyl pyrophosphate synthase (FPS). FPP is then converted to squalene by squalene synthase (SQS), which is further converted to 2,3-oxidosqualene by squalene epoxidase (SE). Finally, lanosterol synthase (LSS) catalyzes the formation of lanosterol from 2,3-oxidosqualene. The enzyme structures involved in the MVA pathway are highly conserved, and the corresponding genes have been identified and characterized in *G. lucidum*. There are two copies of AACT and FPS genes in the *G. lucidum* genome, while the remaining nine enzymes are encoded by single-copy genes [13].

The downstream biosynthetic pathway in the synthesis of GAs has not been fully identified yet, although several CYP450s have been reported to be involved in the modification of lanosterol and its derivates. In 2012, the genome map of *G. lucidum* was released, revealing 219 CYP450s, including 197 completed genes and 22 pseudogenes [13]. During the transition from mycelium to the primordial stage, 78 out of 197 CYP450 genes were found to be up-regulated; however, these genes were then down-regulated from the primordial stage to the fruiting body, consistent with the accumulation of GAs. This provided a foundation and direction for identifying the key CYP450 involved in GA biosynthesis. Until now, four CYP450 enzymes capable of catalyzing the synthesis of GAs have been identified, including CYP5150L8 [55], CYP512U6 [56], CYP5139G1 [57], and CYP512W2 [17].

CYP5150L8 catalyzes the oxidation of the C-26 position of lanosterol, resulting in the formation of a carboxyl group and the production of 3-hydroxy-lanosta-8,24-dien-26-oic acid (HLDOA). Whereafter, researchers achieved heterologous production of HLDOA by expressing CYP5150L8 in *S. cerevisiae*. And then, they identified another CYP450 enzyme, CYP5139G1, which is responsible for the C-28 oxidation of HLDOA, resulting in the production of a new GA, 3,28-dihydroxy-lanosta-8,24-dien-26-oic acid (DHLDOA). CYP512U6 plays a role in hydroxylating GA-DM and GA-TR at the C-23 position, resulting in the formation of hainanic acid A and GA-Jc, respectively. Additionally, CYP512U6 can hydroxylate a modified form of GA-DM, where the C-3 ketone has been converted to a hydroxyl group by the sterol reductase ERG27 from *S. cerevisiae*. The CYP450 enzymes, CYP5150L8, CYP512U6, and CYP5139G1, were identified as producing type I GA; however, type II GAs usually have more complex modifications. The discovery of CYP512W2 clarified the possible transformation pathway from type I to type II GA. CYP512W2 has the ability to hydroxylate at either C-7 or C-11 of HLDOA, and CYP512W2 could also catalyze the conversion of HLDOA into type II GAs, GA-Y and GA-Jb. These findings have led researchers to propose that type I GA undergoes an elimination reaction, followed by spontaneous electron transfer, ultimately resulting in the formation of a conjugated double bond. The diverse biosynthesis of GAs, particularly the oxidative modification, still needs to be further studied through omics mining and biochemical assays.

## Heterologous synthesis of active ingredients in yeast

The first report on heterologous biosynthesis of GAs involved the expression of CYP5150L8 to produce HLDOA in *S. cerevisiae* [55]. Heterologous biosynthesis of HLDOA was achieved in the engineered yeast YL-T3-CYP5150L8, with a final titer of 14.5 mg/L at 120 h of fermentation. Furthermore, the identification and expression of CYP5139G1 in the YL-T3-CYP5150L8 engineered yeast to achieve the production of DHLDOA, with a yield of 2.2 mg/L [57]. Although the targeted GA biosynthesis has not been fully elucidated, these studies provide valuable insights for the development of heterologous cell factories to produce GAs. LZ-8, a fungal immunomodulatory protein from *G. lucidum*, could treat cancer and autoimmune diseases [58–60]. The crystal structure of LZ-8 from *G. lucidum* has been solved to understand its medicine activity [61]. The in vitro production of recombinant LZ-8 (rLZ-8) using the *Pichia* system has been achieved, and the yield reached up to 270 mg/L [62]. Heterologous biosynthesis of GAs and LZ-8 in yeast, notably *S. cerevisiae* and *P. pastoris*, has emerged as an attractive and efficient method for their green production. These studies provide valuable insights for the development of heterologous cell factories, offering a promising avenue for large-scale production of GAs.

## Deep fermentation of *G. lucidum* mycelia for GA production

GAs are traditionally extracted from the fruiting bodies and spores of the mushroom-producing host, *G. lucidum*. However, cultivating the mushroom in the field is a difficult and time-consuming process, which makes it challenging to ensure consistent product quality. In recent years, a biotechnological approach known as submerged fermentation of *G. lucidum* mycelia has emerged as a promising alternative for GA production [63]. Compared to those found in the fruiting body, the GAs from the mycelium have a greater variety and exhibits superior pharmacological activities [64]. By optimizing the fermentation process, it is possible to significantly improve the production of GAs. Factors that affect liquid fermentation include the screening of *G. lucidum* strains, the composition of culture media [65], pH levels, dissolved oxygen, temperature, and agitation rate [66].

Before GA fermentation, it is necessary to evaluate the growth rate, mycelium yield, and GA content of different strains to identify those with high potential for GA production [67]. Extensive research has been conducted to optimize fermentation parameters, including mycelial biomass and intracellular triterpene yield. For instance, the optimized conditions entailed a malt juice concentration of 4.1%, yeast extract concentration of 1.89%, and pH of 5.40, resulting in an optimal mycelial biomass of 1.87 g/100 mL and intracellular triterpene yield of 93.21 mg/100 mL [63]. Furthermore, studies have reported the optimal fermentation conditions as an initial pH of 5.9, 20.0% dissolved oxygen, and a temperature of 28.6 °C [68]. These conditions led to a triterpene acid yield of 308.1 mg/L in a 5-L stirred bioreactor. Notably, the optimized conditions were successfully scaled up to a production scale of 200 L, achieving a maximum triterpene acid production of 295.3 mg/L and productivity of 49.2 mg/L/day. These results signify a remarkable increase of 80.9% and 111.5%, respectively, compared to the non-optimized conditions. Not only does this method aim to increase the overall triterpene content, but it also enables the enhancement of specific GAs. For example, a response surface methodology was employed to establish a model correlating the production of GA-Me with glucose, peptone, and cultivation time [69]. The model predicted maximum yields of 11.9 mg/L for glucose, 44.4 g/L for peptone, and 437.1 h for cultivation time. Experimental validation confirmed a maximum yield of 12.4 mg/L, representing a remarkable improvement of 129.6% compared to the non-optimized conditions.

There are special methods that can increase GA yields, such as two-stage cultivation [70], three-stage culture process [71], chemical transformation [72], and supplemental nutrient inducing [73–79]. Two-stage cultivation involves shaking flasks for rapid lanosterol accumulation and liquid static cultivation to convert it into GAs, leading to a significant increase in GA yield from 1.36 mg/100 mg DW to 3.19 mg/100 mg DW [70]. A three-stage light strategy was developed, which includes 2 days of dark cultivation followed by 6 days of cultivation under 0.94 W/m^2^ white light irradiation and finally cultivation under 4.7 W/m^2^ white light irradiation until fermentation is completed leading to a maximum GA yield of 3.1 ± 0.1 mg/100 mg DW, significantly higher than dark cultivation and light irradiation cultivation [71]. Another strategy for significantly increasing the yield of the target GA is through the chemical transformation of challenging-to-separate impurities into the desired product, allowing for converting these impurities into the desired GA, thereby enhancing its production [72]. In addition, the supplemental nutrient inducing including microcrystalline cellulose (MCC) and D-galactose, oleic acid, methyl jasmonate (MeJA), and metal ions have been reported to effectively improve GA production in *G. lucidum* mecylia fermentation. For example, adding microcrystalline cellulose (MCC) and d-galactose increased GA production by 85.96% and 63.90%, respectively [78]; oleic acid supplementation during fermentation enhanced the production of GA-R, GA-S, and GA-T [79]; MeJA leads to a 28.6% increase in GA yields [73]; the addition of metal ions such as Mn^2+^ [75], Cu^2+^ [74], and Ca^2+^ [75, 77] showed significant improvements in GA production.

## *Ganoderma lucidum* as medicinal model fungi

In 2012 [13], it was first reported that the *G. lucidum* genome is approximately 43.3 Mb in size and consists of 13 chromosomes encoding 16,113 predicted genes, including various cytochrome P450s, transporters, and regulatory factors related to secondary metabolite synthesis, transport, and regulation. The elucidation of the *G. lucidum* genome makes this organism a potential model system for the study of secondary metabolic pathways and their regulation in medicinal fungi. To date, five different *G. lucidum* genome sequences, one mitochondrial genome, and nearly 200 transcriptome data have been published on NCBI. These omic data have identified abundant CYP450-encoding genes, as well as genes encoding glycosyltransferases and glycoside hydrolases, and a large number of carbohydrate-active enzymes and ligninolytic enzymes. On the other hand, elucidating the structure and function of the complete mitochondrial genome is important to fully understand the genetic contents of *G. lucidum*. The mitochondrial genome is a typical circular DNA molecule of 60,630 bp with a GC content of 26.67% [80]. Genome annotation identified genes that encode 15 conserved proteins, 27 tRNAs, small and large rRNAs, four homing endonucleases, and two hypothetical proteins. The results contribute to the understanding of the functions and evolution of fungal mitochondrial DNA. *G. lucidum* also has a large amount of transcriptome data, including induction by inducers (PRJNA865720), metal ions (PRJNA427373), heat stress (PRJNA419168), and different periods (PRJNA796760) and tissues (PRJNA822528). These transcriptome data provide comprehensive and in-depth gene expression information, which can help elucidate the function of *G. lucidum* genes and further explore the regulatory mechanisms and biological processes.

## Cultivation techniques and genetic transformation system

**Fig. 4:**
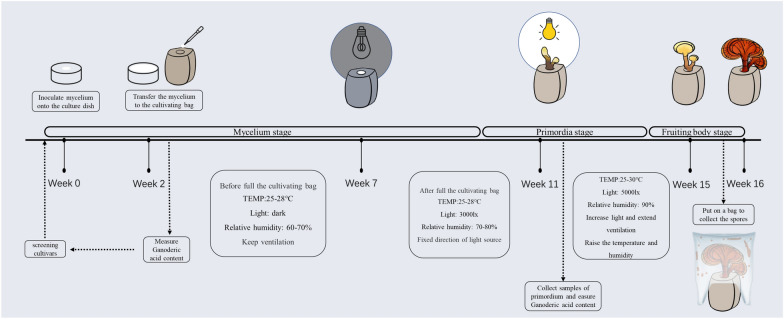
The optimal growth environment for *Ganoderma lucidum* varies across its life cycle stages: mycelium, primordium, fruiting body, and spore production

The successful cultivation of *G. lucidum* using the “spore separation cultivation method” by a Chinese technician in 1970 opened up the possibility of its artificial cultivation in China. Until now, there have been various cultivation methods, including log cultivation, sawdust cultivation, substitute cultivation, outdoor cultivation, indoor cultivation, and biomimetic wild cultivation. The cultivation process of *G. lucidum* can be divided into two stages: mycelial cultivation and fruiting body cultivation [81]. Mycelial cultivation involves the inoculation of mycelium onto liquid or solid media supplemented with requisite nutrients, hormones, and vitamins, followed by sterilization. Upon full colonization, the mycelium is transferred to a cultivation bag containing a substrate blend. Optimal environmental parameters, including controlled temperatures (22–25 °C during colonization, increased to 25–28 °C for accelerated growth), specific humidity levels (60–70% during fruiting, elevated to 90–95% for primordia development), and low light conditions, are crucial throughout the cultivation stages. During spore release, fruiting bodies are enclosed in paper bags secured with rubber bands, requiring a controlled environment of 24 °C temperature, 85% humidity, and adequate ventilation. The subsequent process of bagging and collecting spore powder spans approximately 1 month, culminating in the careful removal of the paper bag during harvesting and the gentle brushing of spore powder into containers prior to fruit body detachment (Fig. 4).

Currently, many researchers have initiated genetic transformation studies on *G. lucidum*. In 2012, using the electroporation method, researchers successfully transferred the GUS and GFP reporter genes driven by the *G. lucidum* GDP promoter into the protoplasts, with the bialaphos resistance gene as the selection marker [66]. Subsequently, the researchers used the PEG-mediated method to transfer the GUS reporter gene into the protoplasts, with the hygromycin resistance gene as the selection marker, at a transformation frequency of about 5–6 transformants/107 protoplasts [63]. In addition, it was found that the protoplasts could be infected using Agrobacterium-mediated transformation, and the endogenous GDP promoter could drive the reporter gene, with the hygromycin resistance gene as the selection marker, resulting in transgenic *G. lucidum* [68]. The transformation efficiency was 200 transformants/105 protoplasts. In 2012, using a mutated succinate dehydrogenase (sdhB) gene as the selection marker, researchers used the herbicide carboxin as a screening pressure to improve the safety of transgenic *G. lucidum* [69]. As these developments have unfolded, the genetic system of *G. lucidum* has undergone significant maturation, offering substantial technical support for the field of functional genomics research.

## Conclusion and future perspectives

*Ganoderma lucidum*, renowned for its extensive pharmacological activities, has gained recognition as a “therapeutic fungal bio-factory,” showcasing its potential as a promising candidate for advancing synthetic biology investigations. This exceptional fungus possesses a diverse repertoire of regulatory elements and synthetic enzyme genes, which hold immense promise in developing an efficient chassis system capable of synthesizing specific secondary metabolites through genome modification and deletion techniques. By delving into the pathways of secondary metabolism and studying the regulatory elements within *G. lucidum*, we can not only address the current scarcity of essential elements in natural drug synthetic biology studies but also unlock the possibility of large-scale production of existing natural drugs to cater to the increasing demand across various domains. Moreover, the ability to reconstruct and modify the biosynthetic pathways of natural drugs within *G. lucidum* provides a platform for the creation of innovative pharmaceutical compounds.

The inherent biological characteristics and well-established research foundation of *G. lucidum* provide a solid basis for its potential as a model organism in medicinal research. However, further investigations into its biology are necessary to fully capitalize on its role as a model. The completion of a refined genome map for *G. lucidum* offers a robust foundation for studying its functional genomics. Moreover, enhancing the genetic transformation system of *G. lucidum* will enable the integration of mainstream biological techniques, including site-directed mutagenesis, gene deletion, and mutagenesis library construction. Additionally, the existing classification system of *Ganoderma* remains a subject of controversy, and the complexities surrounding *G. lucidum* germplasm resources have posed significant challenges to research in this field. Thus, establishing a standardized and widely applicable model organism of *G. lucidum* represents a crucial step toward the standardization and normalization of medicinal model organism research involving *G. lucidum*.

The biosynthesis of GA still faces challenges, but advanced techniques such as T2T genome, single-cell transcriptome, and mass spectrometry imaging can greatly improve the elucidation efficiency. Therefore, the applications of novel strategies in GA biosynthesis need to be further in-depth studied. The biosynthesis pathway of GA will provide important insights for metabolic engineering and molecular breeding of high-quality *G. lucidum* variations.

### Supplementary Information

**Additional file 1: Figure S1.** Chemical structure of triterpenes isolated from *G. lucidum*. **Table S1.** Compound names, molecular formula and literature listings of triterpenes isolated from *G. lucidum*. **Table S2.** Heterologous synthesis of genes derived from *G. lucidum*. **Table S3.** Pharmacological activities of GAs. **Table S4.** The process of improving the yield of GAs. **Table S5.** Transcriptome data on *G. lucidum* in the NCBI database. **Table S6.** Abbreviated list.

## Data Availability

All data generated during this study are included in this published article and supplementary information files.
