# Supplementary material for: Chemodiversity, pharmacological activity, and biosynthesis of specialized metabolites from medicinal model fungi Ganoderma lucidum
Source: Chin Med. 2024 Mar 22;19:51. doi: 10.1186/s13020-024-00922-0 (PMC10958966; doi:10.1186/s13020-024-00922-0)
Supplement: Supplementary file 1 — Additional file 1: Figure S1. Chemical structure of triterpenes isolated from G. lucidum. Table S1. Compound names, molecular formula and literature listings of triterpenes isolated from G. lucidum. Table S2. Heterologous synthesis of genes derived from G. lucidum. Table S3. Pharmacological activities of GAs. Table S4. The process of improving the yield of GAs. Table S5. Transcriptome data on G. lucidum in the NCBI database. Table S6. Abbreviated list. [file 13020_2024_922_MOESM1_ESM.docx]

**Additional file 1 for**

**Chemodiversity, pharmacological activity, and biosynthesis of specialized metabolites from medicinal model fungi *Ganoderma lucidum***

Du et al.

**Figure S1**

Figure S1. Chemical structure of triterpenes isolated from *G. lucidum*. 3

**Tables S1 to S6**

Table S1. Compound names, molecular formula and literature listings of triterpenes isolated from *G. lucidum.*  9

Table S2. Heterologous synthesis of genes derived from *G. lucidum.* 15

Table S3. Pharmacological activities of GAs. 16

Table S4. The process of improving the yield of GAs. 17

Table S5. Transcriptome data on *G. lucidum* in the NCBI database. 19

Table S6. Abbreviated list. 47


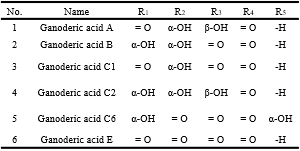

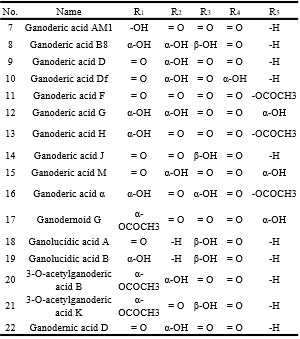

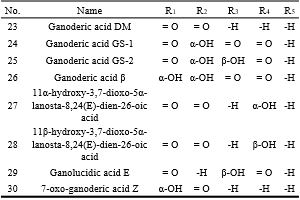

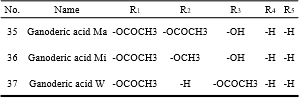

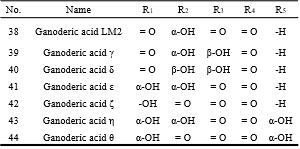

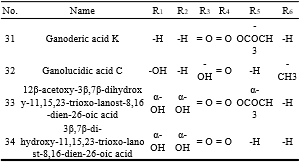

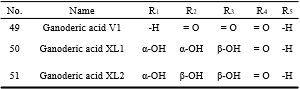

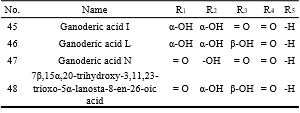

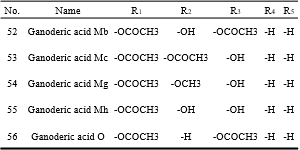


**Type Ⅰ Triterpenoid acid**

**Type Ⅰ Triterpenoid acid**


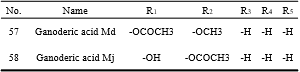

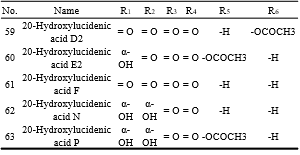

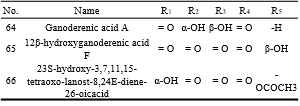

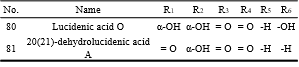

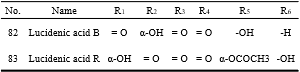

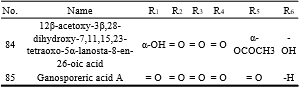

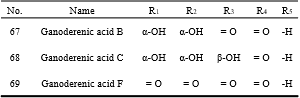

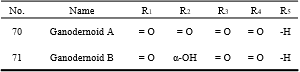

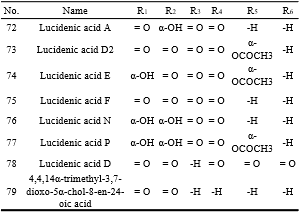

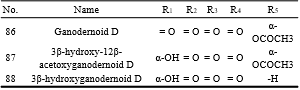


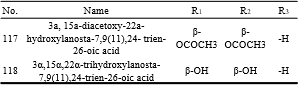

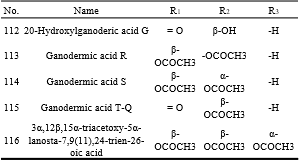

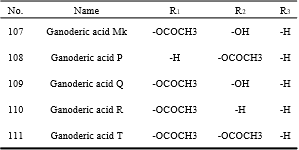

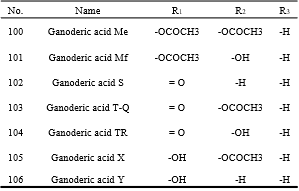


**Type Ⅱ Triterpenoid acid**

**Type Ⅰ Triterpenoid acid**

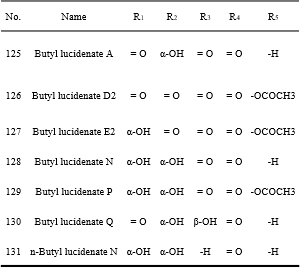

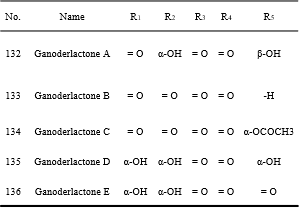

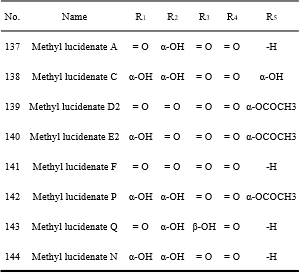


**Type Ⅰ Triterpene ester**


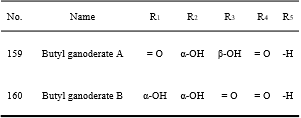

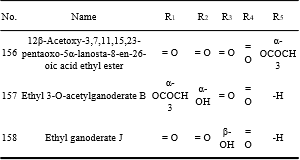

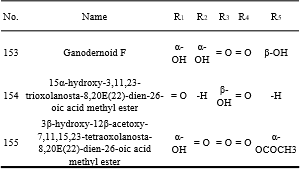

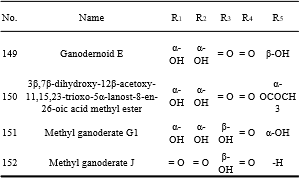

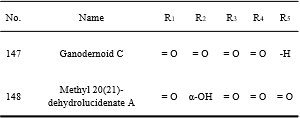

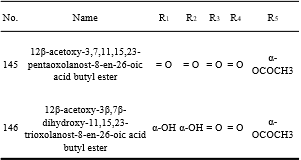


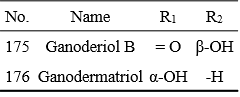

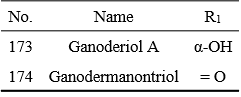

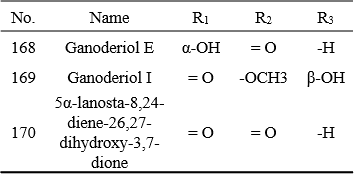

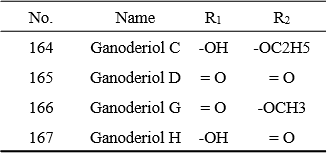


**Type Ⅱ Triterpenol**

**Type Ⅰ Triterpenol**


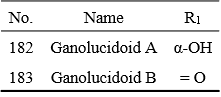

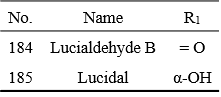

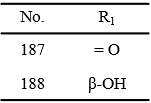

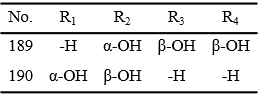

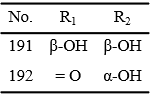


Supplementary figure 1. Chemical structure of triterpenes isolated from *G. lucidum*. The number of each compound corresponds to that in the Supplemental table 1.

**Type Ⅰ Triterpene aldehydes and ketones**

**Others**

**Type Ⅱ Triterpene aldehydes**

Table S1. Compound names, molecular formula and literature listings of triterpenes isolated from *G. lucidum*.

| Supplemental table 1 Compound names, molecular formula and literature listings of triterpenes isolated from *G. lucidum* | | | | |
| --- | --- | --- | --- | --- |
| No. | Name | Molecular formula | Doi | CID |
| 1 | Ganoderic acid A | C30H44O7 | 10.1016/s0031-9422(98)00254-4 | 73554535 |
| 2 | Ganoderic acid B | C30H44O7 | 10.1248/cpb.46.1607 | 471003 |
| 3 | Ganoderic acid C1 | C30H42O7 | TW-586931-B | 471004 |
| 4 | Ganoderic acid C2 | C30H46O7 | 10.1248/cpb.34.3695 | 57396771 |
| 5 | Ganoderic acid C6 | C30H42O8 | 10.1016/j.ejmech.2005.04.015 | 124488037 |
| 6 | Ganoderic acid E | C30H40O7 | 10.1248/cpb.33.2624 | 23247894 |
| 7 | Ganoderic acid AM1 | C30H42O7 | 10.1016/S0031-9422(00)83121-0 | 10346401 |
| 8 | Ganoderic acid B8 | C30H46O7 | EP-0943006-B1 | 21632956 |
| 9 | Ganoderic acid D | C30H42O7 | 10.1248/cpb.33.2624 | 14109406 |
| 10 | Ganoderic acid Df | C30H44O7 | 10.1016/j.fitote.2010.06.025 | 57402147 |
| 11 | Ganoderic acid F | C32H42O9 | 10.1248/cpb.33.2624 | 23247895 |
| 12 | Ganoderic acid G | C30H44O8 | 10.1248/cpb.33.2628 | 20055988 |
| 13 | Ganoderic acid H | C32H44O9 | 10.1248/cpb.33.2624 | 20055989 |
| 14 | Ganoderic acid J | C30H42O7 | 10.1080/00021369.1985.10867324 | 20055991 |
| 15 | Ganoderic acid M | C30H42O8 | 10.1016/j.phytochem.2022.113169 | 5317490 |
| 16 | Ganoderic acid α | C32H46O9 | 10.1016/s0031-9422(98)00254-4 | 471001 |
| 17 | Ganodernoid G | C32H44O9 | 10.1021/acs.jnatprod.5b00132 | 139587706 |
| 18 | Ganolucidic acid A | C30H44O6 | 10.1248/cpb.33.2628 | 20055993 |
| 19 | Ganolucidic acid B | C30H46O6 | 10.1248/cpb.33.2628 | 20055994 |
| 20 | 3-O-acetylganoderic acid B | C32H46O8 | 10.1002/hlca.200900028 | 101491698 |
| 21 | 3-O-acetylganoderic acid K | C32H46O8 | 10.1002/hlca.200900028 | 101491701 |
| 22 | Ganodernic acid D | C30H42O7 | 10.1248/cpb.33.4829 | 14109406 |
| 23 | Ganoderic acid DM | C30H44O4 | 10.5281/zenodo.3541258 | 11784642 |
| 24 | Ganoderic acid GS-1 | C30H42O6 | 10.1016/j.jpba.2021.114355 | 44473462 |
| 25 | Ganoderic acid GS-2 | C30H44O6 | 10.1016/j.jpba.2021.114355 | 44473463 |
| 26 | Ganoderic acid β | C30H44O6 | 10.1248/cpb.46.1607 | 10097521 |
| 27 | 11α-hydroxy-3,7-dioxo-5α-lanosta-8,24(E)-dien-26-oic acid | C30H44O5 | 10.1016/j.phytochem.2010.06.005 | 46910042 |
| 28 | 11β-hydroxy-3,7-dioxo-5α-lanosta-8,24(E)-dien-26-oic acid | C30H44O5 | 10.1016/j.phytochem.2010.06.005 | 46910043 |
| 29 | Ganolucidic acid E | C30H44O5 | 10.1080/00021369.1988.10868655 | 15602283 |
| 30 | 7-oxo-ganoderic acid Z | C30H46O4 | 10.1080/14786410600921466 | 71461154 |
| 31 | Ganoderic acid K | C32H46O9 | 10.1248/cpb.34.3025 | 74036828 |
| 32 | Ganolucidic acid C | C30H46O7 | 10.1080/00021369.1985.10867324 | 14109400 |
| 33 | 12β‐acetoxy‐3β,7β‐dihydroxy‐11,15,23‐trioxo‐lanost‐8,16‐dien‐26‐oic acid | C32H44O9 | 10.1002/mrc.2046 | 139585069 |
| 34 | 3β,7β‐di‐ hydroxy‐11,15,23‐trioxo‐lanost‐8,16‐dien‐26‐oic acid | C30H42O7 | 10.1002/mrc.2046 | 139586408 |
| 35 | Ganoderic acid Ma | C34H52O7 | 10.1271/bbb1961.51.619 | 131751707 |
| 36 | Ganoderic acid Mi | C33H52O6 | 10.1271/bbb1961.51.1149 | 131751712 |
| 37 | Ganoderic acid W | C30H52O7 | 10.1271/bbb1961.51.619 | 131751368 |
| 38 | Ganoderic acid LM2 | C30H42O7 | 10.1016/j.ejmech.2019.04.039 | 11813266 |
| 39 | Ganoderic acid γ | C30H44O7 | 10.1248/CPB.48.1026 | 15427808 |
| 40 | Ganoderic acid δ | C30H44O7 | 10.1248/CPB.48.1026 | 15427809 |
| 41 | Ganoderic acid ε | C30H44O7 | 10.1248/CPB.48.1026 | 15427810 |
| 42 | Ganoderic acid ζ | C30H42O7 | 10.1248/CPB.48.1026 | 102306794 |
| 43 | Ganoderic acid η | C30H44O8 | 10.1248/CPB.48.1026 | 10721020 |
| 44 | Ganoderic acid θ | C30H42O8 | 10.1248/CPB.48.1026 | 15427811 |
| 45 | Ganoderic acid I | C30H44O8 | 10.1248/cpb.33.2628 | 20055990 |
| 46 | Ganoderic acid L | C30H46O8 | CN-113121631-A | 101600071 |
| 47 | Ganoderic acid N | C30H42O8 | 10.1016/j.jpba.2021.114355 | 131751706 |
| 48 | 7β,15α,20-trihydroxy-3,11,23-trioxo-5α-lanosta-8-en-26-oic acid | C30H44O8 | 10.1021/acs.jafc.9b01195 | 146682729 |
| 49 | Ganoderic acid V1 | C30H42O7 | 10.1016/j.jpba.2021.114355 | 131752702 |
| 50 | Ganoderic acid XL1 | C30H45O5 | 10.1016/j.fitote.2014.08.004 |  |
| 51 | Ganoderic acid XL2 | C30H45O5 | 10.1016/j.fitote.2014.08.004 |  |
| 52 | Ganoderic acid Mb | C36H54O9 | 10.1271/bbb1961.51.619 | 13916716 |
| 53 | Ganoderic acid Mc | C36H54O9 | 10.1271/bbb1961.51.619 | 131751855 |
| 54 | Ganoderic acid Mg | C35H54O8 | 10.1271/bbb1961.51.1149 | 131751896 |
| 55 | Ganoderic acid Mh | C34H52O8 | 10.1271/bbb1961.51.1149 |  |
| 56 | Ganoderic acid O | C36H54O9 | 10.1016/j.ejmech.2019.04.039 |  |
| 57 | Ganoderic acid Md | C35H54O7 | 10.1271/bbb1961.51.619 | 131751708 |
| 58 | Ganoderic acid Mj | C33H52O6 | 10.1271/bbb1961.51.1149 | 131751722 |
| 59 | 20-Hydroxylucidenic acid D2 | C28H36O9 | 10.1021/np040230h | 139585728 |
| 60 | 20-Hydroxylucidenic acid E2 | C29H40O9 | 10.1021/np040230h | 11443981 |
| 61 | 20-Hydroxylucidenic acid F | C27H36O7 | 10.1021/np040230h | 11454258 |
| 62 | 20-Hydroxylucidenic acid N | C27H40O7 | 10.1021/np040230h | 11408923 |
| 63 | 20-Hydroxylucidenic acid P | C29H42O9 | 10.1021/np040230h | 11191849 |
| 64 | Ganoderenic acid A | C30H42O7 | 10.1016/0031-9422(89)85036-8 | 14193987 |
| 65 | 12β-hydroxyganoderenic acid F | C30H38O8 | 10.1016/j.phytol.2016.03.007 | 139587802 |
| 66 | 23S-hydroxy-3,7,11,15-tetraoxo-lanost-8,24E-diene-26-oicacid | C30H40O7 | 10.1080/10286020802016297 | 25000689 |
| 67 | Ganoderenic acid B | C30H42O7 | 10.1248/cpb.33.4829 | 139584158 |
| 68 | Ganoderenic acid C | C30H44O7 | 10.1248/cpb.33.4829 | 139584921 |
| 69 | Ganoderenic acid F | C30H38O7 | 10.1248/cpb.33.4829 | 139585360 |
| 70 | Ganodernoid A | C25H32O6 | 10.1021/acs.jnatprod.5b00132 | 122184972 |
| 71 | Ganodernoid B | C25H34O6 | 10.1021/acs.jnatprod.5b00132 | 139585120 |
| 72 | Lucidenic acid A | C27H38O6 | 10.1016/j.phytochem.2010.06.005 | 14109375 |
| 73 | Lucidenic acid D2 | C29H38O8 | 10.1248/cpb.33.2624 | 23247891 |
| 74 | Lucidenic acid E | C29H40O8 | 10.1248/cpb.33.2624 | 23247892 |
| 75 | Lucidenic acid F | C27H36O6 | 10.1248/cpb.33.2624 | 146684986 |
| 76 | Lucidenic acid N | C27H40O6 | 10.1021/np010115w | 21592283 |
| 77 | Lucidenic acid P | C29H42O8 | 10.1021/np0302293 | 11203160 |
| 78 | Lucidenic acid D | C27H34O7 | 10.1248/cpb.33.4829 | 139585468 |
| 79 | 4,4,14α-trimethyl-3,7-dioxo-5α-chol-8-en-24-oic acid | C27H40O4 | 10.1016/j.phytochem.2010.06.005 | 46910045 |
| 80 | Lucidenic acid O | C27H40O7 | 10.1016/s0968-0896(99)00121-2 | 9847846 |
| 81 | 20(21)-dehydrolucidenic acid A | C27H36O6 | 10.1021/np040230h | 11340050 |
| 82 | Lucidenic acid B | C27H38O7 | 10.1271/bbb1961.48.2905 | 139585087 |
| 83 | Lucidenic acid R | C29H40O9 | 10.1021/acs.jafc.9b01195 | 146682725 |
| 84 | 12β-acetoxy-3β,28-dihydroxy-7,11,15,23-tetraoxo-5α-lanosta-8-en-26-oic acid | C32H44O10 | 10.1021/acs.jafc.9b01195 | 146682728 |
| 85 | Ganosporeric acid A | C30H38O8 | 10.1615/intjmedmushrooms.v1.i2.40 | 139583540 |
| 86 | Ganodernoid D | C32H40O9 | 10.1021/acs.jnatprod.5b00132 | 139587039 |
| 87 | 3β-hydroxy-12β-acetoxyganodernoid D | C32H42O9 | 10.1016/j.phytol.2016.03.007 | 139583547 |
| 88 | 3β-hydroxyganodernoid D | C30H40O7 | 10.1016/j.phytol.2016.03.007 | 139585928 |
| 89 | Ganoderic acid U | C30H48O4 | CN-101747400-B | 101600072 |
| 90 | Ganoderic acid Σ | C30H44O7 | 10.1016/j.tetlet.2016.10.072 | 139586441 |
| 91 | Ganolucidic acid D | C30H44O6 | 10.1080/00021369.1986.10867474 | 122201289 |
| 92 | Lucidenic acid C | C27H42O6 | 10.1248/cpb.33.4829 | 139588461 |
| 93 | 12β-Acetoxy-7β-hydroxy-3,11,15,23-tetraoxo-5α-lanosta-8,20-dien-26-oic acid | C32H42O9 | 10.1016/j.phytochem.2010.06.005 | 46910044 |
| 94 | 12β-acetoxyganoderic acid θ | C32H44O9 | 10.1021/acs.jafc.9b01195 | 146682730 |
| 95 | 3b,15a-Diacetoxylanosta-8,24-dien-26-oic acid | C34H52O6 | 10.1021/np50059a017 | 139585073 |
| 96 | 23S-hydroxy-3,7,11,15-tetraoxo-lanost-8,24E-diene-26-oicacid | C30H40O7 | 10.1080/10286020802016297 | 25000689 |
| 97 | 7-O-ethyl ganoderic acid O | C38H58O9 | 10.1080/10286020.2010.493506 | 46894364 |
| 98 | Ganoderic acid V | C32H48O6 | 10.1126/science.1174621 | 131751369 |
| 99 | Ganoderic acid AP3 | C30H42O8 | 10.1248/cpb.56.1035 | 101863399 |
| 100 | Ganoderic acid Me | C34H50O6 | 10.1007/s00253-020-10476-4 | 13969079 |
| 101 | Ganoderic acid Mf | C32H48O5 | 10.1271/bbb1961.51.619 | 14137634 |
| 102 | Ganoderic acid S | C30H44O3 | 10.1248/cpb.34.2282 | 12444571 |
| 103 | Ganoderic acid T-Q | C32H46O5 | 10.1021/np50059a017 | 10436380 |
| 104 | Ganoderic acid TR | C30H44O4 | 10.1016/j.bmc.2006.08.018 | 139584401 |
| 105 | Ganoderic acid X | C32H48O5 | 10.3109/14756366.2012.658787 | 14137626 |
| 106 | Ganoderic acid Y | C30H46O3 | 10.1016/j.jep.2017.08.041 | 57397445 |
| 107 | Ganoderic acid Mk | C34H50O7 | 10.1271/bbb1961.51.1149 | 13916707 |
| 108 | Ganoderic acid P | C34H50O7 | 10.1016/j.ejmech.2019.04.039 |  |
| 109 | Ganoderic acid Q | C34H50O7 | 10.1016/j.ejmech.2019.04.039 |  |
| 110 | Ganoderic acid R | C34H50O6 | 10.1248/cpb.34.2282 | 21637706 |
| 111 | Ganoderic acid T | C36H52O8 | 10.1248/cpb.34.2282 | 21637704 |
| 112 | 20-Hydroxylganoderic acid G | C30H40O3 | 10.1021/np100031c | 156580517 |
| 113 | Ganodermic acid R | C34H50O6 | 10.1021/np50053a019 | 9985134 |
| 114 | Ganodermic acid S | C34H50O6 | 10.1021/np50053a019 | 139586622 |
| 115 | Ganodermic acid T-Q | C32H46O5 | 10.1021/NP50059A017 | 10436380 |
| 116 | 3α,12β,15α-triacetoxy-5α-lanosta-7,9(11),24-trien-26-oic acid | C36H52O8 | 10.1016/j.phytol.2015.02.012 | 139583909 |
| 117 | 3a, 15a-diacetoxy-22a-hydroxylanosta-7,9(11),24- trien-26-oic acid | C34H50O7 | 10.1021/np50059a017 | 139583632 |
| 118 | 3α,15α,22α-trihydroxylanosta-7,9(11),24-trien-26-oic acid | C30H46O5 | 10.1021/np50059a017 | 139584254 |
| 119 | Ganoderic acid Jc | C30H44O5 | 10.1016/j.jpba.2021.114355 | 139585892 |
| 120 | Ganoderic acid SZ | C30H44O3 | 10.1248/cpb.34.2282 | 46888171 |
| 121 | Ganodermic acid Jb | C30H46O4 | 10.1016/j.ejmech.2019.04.039 | 14325145 |
| 122 | Ganodermic acid P2 | C34H50O7 | 10.1016/j.ejmech.2019.04.039 | 13916711 |
| 123 | 22b-Acetoxy-3a,15a-trihydroxylanosta-7,9(11),24-trien-26-oic acid | C32H48O6 | 10.1021/np50059a017 | 139585392 |
| 124 | 23-hydroxyganoderic acid S | C30H46O4 | 10.1021/np100031c | 156580336 |
| 125 | Butyl lucidenate A | C31H46O6 | 10.1021/np900578h | 46184564 |
| 126 | Butyl lucidenate D2 | C33H46O8 | 10.1016/j.bmcl.2012.12.066 | 139586782 |
| 127 | Butyl lucidenate E2 | C33H48O8 | 10.1016/j.bmcl.2012.12.066 | 71582787 |
| 128 | Butyl lucidenate N | C31H48O6 | 10.1021/np900578h | 46184563 |
| 129 | Butyl lucidenate P | C33H50O8 | 10.1016/j.bmcl.2012.12.066 | 71582786 |
| 130 | Butyl lucidenate Q | C31H48O6 | 10.1016/j.bmcl.2012.12.066 | 71582788 |
| 131 | n-Butyl lucidenate N | C31H50O5 | 10.1021/np900578h | 57390352 |
| 132 | Ganoderlactone A | C27H36O7 | 10.1021/acs.jnatprod.5b00132 | 122184969 |
| 133 | Ganoderlactone B | C27H34O6 | 10.1021/acs.jnatprod.5b00132 | 122184973 |
| 134 | Ganoderlactone C | C29H36O8 | 10.1021/acs.jnatprod.5b00132 | 139585165 |
| 135 | Ganoderlactone D | C27H38O7 | 10.1021/acs.jnatprod.5b00132 | 122184970 |
| 136 | Ganoderlactone E | C27H36O7 | 10.1021/acs.jnatprod.5b00132 | 122184971 |
| 137 | Methyl lucidenate A | C28H40O6 | 10.1016/J.BMCL.2010.06.093 | 21636089 |
| 138 | Methyl lucidenate C | C28H42O7 | 10.1248/CPB.34.3695 | 11569423 |
| 139 | Methyl lucidenate D2 | C30H40O8 | 10.1248/CPB.33.2624 | 21633083 |
| 140 | Methyl lucidenate E2 | C30H42O8 | 10.1016/J.BMCL.2010.06.093 | 21633084 |
| 141 | Methyl lucidenate F | C28H38O6 | 10.1021/np010115w | 15508108 |
| 142 | Methyl lucidenate P | C30H44O8 | 10.1021/np0302293 | 11203394 |
| 143 | Methyl lucidenate Q | C28H42O6 | 10.1021/np0302293 | 11271456 |
| 144 | Methyl lucidenate N | C28H42O6 | 10.1080/10286020.2013.820712 | 71718315 |
| 145 | 12β-acetoxy-3,7,11,15,23-pentaoxolanost-8-en-26-oic acid butyl ester | C36H50O9 | 10.1002/cbdv.201400004 | 139586706 |
| 146 | 12β-acetoxy-3β,7β-dihydroxy-11,15,23-trioxolanost-8-en-26-oic acid butyl ester | C36H54O9 | 10.1002/cbdv.201400004 | 86279796 |
| 147 | Ganodernoid C | C28H36O6 | 10.1021/acs.jnatprod.5b00132 | 139584993 |
| 148 | Methyl 20(21)-dehydrolucidenate A | C28H38O6 | 10.1021/np040230h | 11155641 |
| 149 | Ganodernoid E | C31H46O8 | 10.1021/acs.jnatprod.5b00132 | 139588234 |
| 150 | 3β,7β-dihydroxy-12β-acetoxy-11,15,23-trioxo-5α-lanost-8-en-26-oic acid methyl ester | C33H48O9 | 10.1016/j.phytochem.2010.06.005 | 46910126 |
| 151 | Methyl ganoderate G1 | C31H48O8 | 10.3390/molecules24234353 | 146682671 |
| 152 | Methyl ganoderate J | C31H44O7 | 10.1080/00021369.1985.10867324 | 71582789 |
| 153 | Ganodernoid F | C31H44O8 | 10.1021/acs.jnatprod.5b00132 | 139586712 |
| 154 | 15α-hydroxy-3,11,23-trioxolanosta-8,20E(22)-dien-26-oic acid methyl ester | C31H44O6 | 10.1016/j.phytochem.2019.112256 | 156582654 |
| 155 | 3β-hydroxy-12β-acetoxy-7,11,15,23-tetraoxolanosta-8,20E(22)-dien-26-oic acid methyl ester | C33H44O9 | 10.1016/j.phytochem.2019.112256 | 156582653 |
| 156 | 12β-Acetoxy-3,7,11,15,23-pentaoxo-5α-lanosta-8-en-26-oic acid ethyl ester | C34H46O9 | 10.1016/j.phytochem.2010.06.005 | 46910046 |
| 157 | Ethyl 3-O-acetylganoderate B | C34H50O8 | 10.1002/hlca.200900028 | 101491697 |
| 158 | Ethyl ganoderate J | C32H46O7 | 10.1002/hlca.200900028 | 101491700 |
| 159 | Butyl ganoderate A | C34H52O7 | 10.1021/np900578h | 46184561 |
| 160 | Butyl ganoderate B | C34H52O7 | 10.1021/np900578h | 46184562 |
| 161 | Ethyl lucidenate A | C29H42O6 | 10.1080/14786419.2011.652961 | 71665526 |
| 162 | 12-epi-ganoderlactone D | C27H38O7 | 10.1016/j.phytol.2016.03.007 | 139585814 |
| 163 | Methyl ganoderate A acetonide | C34H50O7 | 10.1016/j.bmcl.2011.04.042 | 57402629 |
| 164 | Ganoderiol C | C32H54O5 | 10.1080/00021369.1988.10868655 | 156580390 |
| 165 | Ganoderiol D | C30H48O5 | 10.1080/00021369.1988.10868655 | 139585300 |
| 166 | Ganoderiol G | C31H52O5 | 10.1080/00021369.1988.10868655 | 156580353 |
| 167 | Ganoderiol H | C30H50O5 | 10.1080/00021369.1988.10868655 | 156580445 |
| 168 | Ganoderiol E | C30H48O4 | 10.1080/00021369.1988.10868655 | 15602281 |
| 169 | Ganoderiol I | C31H50O5 | 10.1080/00021369.1988.10868655 | 15602271 |
| 170 | 5α-lanosta-8,24-diene-26,27-dihydroxy-3,7-dione | C30H46O4 | 10.1016/j.phytol.2015.02.012 | 122182467 |
| 171 | Lucidadiol | C30H48O3 | 10.1021/np990295y | 10789991 |
| 172 | Lucidumol A | C30H48O4 | 10.1248/cpb.46.1607 | 475410 |
| 173 | Ganoderiol A | C30H50O4 | 10.1271/bbb1961.50.2887 | 100927467 |
| 174 | Ganodermanontriol | C30H48O4 | 10.1021/np50048a029 | 73177 |
| 175 | Ganoderiol B | C30H46O4 | 10.1271/bbb1961.50.2887 | 471007 |
| 176 | Ganodermatriol | C30H48O3 | 10.1021/np50046a010 | 21124247 |
| 177 | (24S, 25R)-25-methoxylanosta-7,9(11)-dien-3β,24,26-triol | C31H52O4 | 10.1016/j.phytochem.2019.112256 | 156582651 |
| 178 | 26,27-dihydroxy-24,25-epoxylanosta-7,9(11)-dien-3-one | C30H46O4 | 10.1016/j.phytochem.2019.112256 | 156582652 |
| 179 | Ganodermadiol | C30H48O2 | 10.1021/np50046a010 | 139586903 |
| 180 | Ganodermanondiol | C30H48O3 | 10.1021/np50048a029 | 10253565 |
| 181 | Ganodermenonol | C30H46O2 | 10.1021/np50046a010 | 13934284 |
| 182 | Ganolucidoid A | C26H36O7 | 10.1016/j.phytochem.2019.112256 | 156582649 |
| 183 | Ganolucidoid B | C26H34O7 | 10.1016/j.phytochem.2019.112256 | 156582650 |
| 184 | Lucialdehyde B | C30H44O3 | 10.1248/cpb.50.837 | 10343868 |
| 185 | Lucidal | C30H46O3 | 10.1021/np990295y | 10366713 |
| 186 | Ganoderic aldehyde TR | C30H44O3 | 10.1021/NP100031C | 156580517 |
| 187 | 8β,9α-dihydroganoderic acid C | C30H42O7 | 10.1002/hlca.200900028 | 101491699 |
| 188 | 8β,9α-dihydroganoderic acid J | C30H44O7 | 10.1021/np010385e | 139585040 |
| 189 | 22E, 24R-ergosta-7,22-diene-3β,5α,6β,9α,14α-pentol | C28H46O5 | 10.1080/14786410601129721 | 139585605 |
| 190 | Ergosta-7,22-dien-2β,3α,9α-triol | C28H46O3 | 10.1016/j.phytochem.2007.06.008 | 69889633 |
| 191 | Ganodermaside A | C28H40O2 | 10.1016/j.bmc.2009.12.070 | 70696019 |
| 192 | Ganodermaside B | C28H40O2 | 10.1016/j.bmc.2009.12.070 | 46232683 |
| 193 | Methyl lucidenate K | C28H42O6 | 10.1021/acs.jafc.9b01195 | 146682726 |
| 194 | Methyl 8β,9α-dihydroganoderate J | C31H46O7 | 10.1021/np010385e | 139583646 |
| 195 | (24E)-9a,11a-epoxy-3b-hydroxylanosta-7,24-dien-26-al | C30H46O3 | 10.1080/10286020.2014.996139 | 139584065 |
| 196 | 25α-hydroxyergosta-7,22-dien-3-one | C28H44O2 | 10.5281/zenodo.3541358 | 139588565 |

Table S2. Heterologous synthesis of genes derived from *G. lucidum.*

| Supplemental table 2 Heterologous synthesis of genes derived from *G. lucidum* | | | | |
| --- | --- | --- | --- | --- |
| Gene | Host | Product | Function | doi |
| CYP5150L8 | Saccharomyces cerevisiae | HLDOA | Ganoderic acid biosynthesis | 10.1002/bit.26583 |
| CYP5139G1 | Saccharomyces cerevisiae | DHLDOA | Ganoderic acid biosynthesis | 10.1007/s00253-021-11717-w |
| LZ-8 gene | Pichia pastoris | LZ-8 | Immune regulation and promotion of cell mitosis | 10.2323/jgam.54.393 |
| laccase gene | Pichia pastoris | laccase | Catalytic degradation of various pollutants in vitro | 10.1002/jobm.201200808 |

Table S3. Pharmacological activities of GAs.

| Pharmacological activities of GAs | | | | | | | |
| --- | --- | --- | --- | --- | --- | --- | --- |
| Type | Name | Antitumor | Anti-inflammatory | Antioxidant | Reduction of blood fat and blood pressure | Regulation of liver functions | Protective effect on the lung |
| Type Ⅰ | Ganoderic acid A | Yang et al. 2018. | Wang et al. 2021. | Gill et al. 2019 | Zhu et al. 2018 | Xu et al. 2019 | Wen et al. 2020 |
|  | Ganoderic acid AM1 |  |  |  |  | Liu et al. 2014 |  |
|  | Ganoderic acid B |  |  |  |  |  | Shi et al. 2020 |
|  | Ganoderic acid D | Liu et al. 2018 |  | Yuan et al. 2022 |  |  |  |
|  | Ganoderic acid DM | Bryant et al. 2017 |  |  |  |  |  |
|  | Ganoderic acid F | Kimura et al. 2002 | Sheng et al. 2019 |  |  |  |  |
|  | Ganoderic acid K |  |  |  |  | Li et al. 2023 |  |
| Type Ⅱ | Ganoderic acid T | Chen et al. 2010 |  |  |  |  | Tang el al.2006 |
|  | Ganoderic acid X | Li et al. 2005 |  |  |  |  |  |
|  | Ganoderic acid Y | Du et al.2017 |  |  | Hajjaj et al.2005 |  |  |

Table S4. The process of improving the yield of GAs.

| Supplemental table 3 The process of improving the yield of GAs | | | | | | |
| --- | --- | --- | --- | --- | --- | --- |
| Parameter | Additive | Method | Product | Yield/yield rate | Increase rate | doi |
| glucose 44.4 g/L, peptone 5.0 g/L and fermentation time 437.1 h |  |  | GA-Me |  | 129.60% | 10.3390/molecules171112575 |
| Oxygen concentration 80% |  |  | Total GA | 1427.2 +/- 74.2 mg/l |  | 10.1007/s00449-009-0379-9 |
| Initial pH 5.9,DO 20.0% and temperature 28.6℃ |  |  | Total GA | 308.1mg /L in 5L reactor，295.3 mg/L in 200L reacter | 80.9% (200L) | 10.3390/molecules21101395 |
| pH 6.0, Rotational speed 161.9 rpm |  |  | Total GA | 291.0 mg / L in 5L reactor, 47.9 mg /L/d in 200L reacter | 70.8% (5L) | 10.1615/IntJMedMushrooms.v19.i5.40 |
| Wort 4.10% (0.041 g/ml), yeast extract 1.89% (0.0189 g/ml), pH 5.40 |  |  | Total GA | 93.21 mg/100 ml |  | 10.1631/jzus.B1500147 |
| pH 3.0 4d + pH 4.5 6d |  | pH-shift | Total GA | 321.6 mg/L | pH 3.0 (45%) ph 4.5 (300%) | 10.1016/j.biortech.2008.10.005 |
| DOT 25% 6d + DOT 10% 6d |  | DOT-shift | Total GA | 487.1 mg/L | DOT 25 (43%) DOT 10 (230%) | 10.1016/j.biortech.2008.10.005 |
|  |  | pH-shift + DOT-shift | Total GA | 754.6 mg/L |  | 10.1016/j.biortech.2008.10.005 |
|  |  | Liquid superficial-static culture | Total GA | 23.75±0.60 mg/g |  | 10.1111/1751-7915.13670 |
| solid potato dextrose medium |  | solid-medium culture | Total GA |  |  | 10.1271/bbb.120270 |
| hydrolysis-acetylation-hydrolysis |  | chemical conversion strategy | GA-T | 1.621 g/100g dried mycelia | 365% | 10.1002/bit.24358 |
| 4d shake-flask fermentation + 12d static culture |  | Two-stage culture | Total GA | 3.19 mg/100 mg dried mycelia |  | 10.1021/bp010136g |
| 2d dark + 6d 0.94 W/m2 white light + 4.70 W/m2 white light |  | three-stage light irradiation | Total GA | 3.1+/-0.1 mg/100 mg dried mycelia |  | 10.1002/btpr.36 |
|  | oleic acid |  | GA-R, GA-S, GA-T |  | 311%, 519%, 144% | 10.3390/jof8060615 |
|  | wood decaying components |  | Total GA |  | 185.96% (add 1.5% microcrystalline cellulose), 163.9% (add 1.5% 0.5% D-galactose) | 10.1038/srep46623 |
| 254 microM MeJA solubilized in Tween-20， add to the culture on day 6 | methyl jasmonate |  | Total GA | 4.52 mg / 100mg dried mycelia |  | 10.1016/j.biortech.2010.03.118 |
| 10 mM Mn2+ at the beginning of the static liquid culture | Mn2+ |  | Total GA |  | 220% | 10.1002/bit.25288 |
| add each 1 mM Cu2+ on day 2, 6, 8 and 2 mM Cu2+ on day 4 | Cu2+ |  | Total GA |  |  | 10.1002/btpr.352 |
| glucose 55 g/L, yeast extract14 g/L, ferric acid 0.3 g/L | FeSO4 |  | Total GA | 670 g/L in 300L reacter |  | 10.1007/s00449-014-1152-2 |
| 10 Mm calcium ion | Ca2+ |  | GA-MK, GA-T, GA-S, GA-Me |  | 370% (总GAs), 260%(GA Mk)、450%(GA t). 320%(GA s), 380%(GA me) | 10.1016/j.biotechadv.2011.10.001 |
| Nitrogen restriction condition | Ca2+ |  | GA-T | 1.87 mg/ 100 mg dried mycelia |  | 10.1007/s00449-015-1491-7 |

Table S5. Transcriptome data on *G. lucidum* in the NCBI database.

| Supplemental table 4 Transcriptome data on *G. lucidum* in the NCBI database | | | | | | | | | | | | |
| --- | --- | --- | --- | --- | --- | --- | --- | --- | --- | --- | --- | --- |
| Experiment Accession | Experiment Title | Instrument | Submitter | Study Accession | Study Title | Sample Accession | Total Size, Mb | Total Spots | Total Bases | Library Name | Library Source | Library Selection |
| SRX10172313 | RNA-Seq of Ganoderma lucidum: fruiting body with a few of spores | Illumina NovaSeq 6000 | Guangdong Academy of Sciences | SRP308292 | Ganoderma lucidum strain:YW-1 Raw sequence reads | SRS8322149 | 2161.74 | 23623079 | 7083363776 | L5 | TRANSCRIPTOMIC | Oligo-dT |
| SRX10172314 | RNA-Seq of Ganoderma lucidum: fruiting body with a few of spores | Illumina NovaSeq 6000 | Guangdong Academy of Sciences | SRP308292 | Ganoderma lucidum strain:YW-1 Raw sequence reads | SRS8322150 | 1979.42 | 21751686 | 6523005946 | L6 | TRANSCRIPTOMIC | Oligo-dT |
| SRX10172315 | RNA-Seq of Ganoderma lucidum: fruiting body with a lot of spores | Illumina NovaSeq 6000 | Guangdong Academy of Sciences | SRP308292 | Ganoderma lucidum strain:YW-1 Raw sequence reads | SRS8322151 | 1919.46 | 21039237 | 6309488212 | L7 | TRANSCRIPTOMIC | Oligo-dT |
| SRX10172316 | RNA-Seq of Ganoderma lucidum: fruiting body with a lot of spores | Illumina NovaSeq 6000 | Guangdong Academy of Sciences | SRP308292 | Ganoderma lucidum strain:YW-1 Raw sequence reads | SRS8322152 | 2124.62 | 23123589 | 6934618746 | L8 | TRANSCRIPTOMIC | Oligo-dT |
| SRX10172317 | RNA-Seq of Ganoderma lucidum: fruiting body with a lot of spores | Illumina NovaSeq 6000 | Guangdong Academy of Sciences | SRP308292 | Ganoderma lucidum strain:YW-1 Raw sequence reads | SRS8322153 | 1871.12 | 20406461 | 6119246690 | L9 | TRANSCRIPTOMIC | Oligo-dT |
| SRX10172318 | RNA-Seq of Ganoderma lucidum: fruiting body without spore | Illumina NovaSeq 6000 | Guangdong Academy of Sciences | SRP308292 | Ganoderma lucidum strain:YW-1 Raw sequence reads | SRS8322154 | 2087.43 | 22751195 | 6822589694 | L1 | TRANSCRIPTOMIC | Oligo-dT |
| SRX10172319 | RNA-Seq of Ganoderma lucidum: fruiting body without spore | Illumina NovaSeq 6000 | Guangdong Academy of Sciences | SRP308292 | Ganoderma lucidum strain:YW-1 Raw sequence reads | SRS8322155 | 1859.46 | 20328008 | 6095996230 | L2 | TRANSCRIPTOMIC | Oligo-dT |
| SRX10172320 | RNA-Seq of Ganoderma lucidum: fruiting body without spore | Illumina NovaSeq 6000 | Guangdong Academy of Sciences | SRP308292 | Ganoderma lucidum strain:YW-1 Raw sequence reads | SRS8322156 | 2020.73 | 22108455 | 6630127334 | L3 | TRANSCRIPTOMIC | Oligo-dT |
| SRX10172321 | RNA-Seq of Ganoderma lucidum: fruiting body with a few of spores | Illumina NovaSeq 6000 | Guangdong Academy of Sciences | SRP308292 | Ganoderma lucidum strain:YW-1 Raw sequence reads | SRS8322157 | 2085.88 | 22796672 | 6835862842 | L4 | TRANSCRIPTOMIC | Oligo-dT |
| SRX105468 | Illumina sequencing of RNAseq from Ganoderma lucidum strain CGMCC5.0026 | Illumina HiSeq 2000 | IMPLAD | SRP009291 | Ganoderma lucidum G.260125-1 Genome sequencing and assembly | SRS284643 | 4880.44 | 35703741 | 7140748200 | galu_Illumina_RNAseq_11C09094 | TRANSCRIPTOMIC | RANDOM |
| SRX105469 | Illumina sequencing of RNAseq from Ganoderma lucidum strain CGMCC5.0026 | Illumina HiSeq 2000 | IMPLAD | SRP009291 | Ganoderma lucidum G.260125-1 Genome sequencing and assembly | SRS284643 | 3926.22 | 27774625 | 5554925000 | galu_Illumina_RNAseq_11C09095 | TRANSCRIPTOMIC | RANDOM |
| SRX105470 | Illumina sequencing of RNAseq from Ganoderma lucidum strain CGMCC5.0026 | Illumina HiSeq 2000 | IMPLAD | SRP009291 | Ganoderma lucidum G.260125-1 Genome sequencing and assembly | SRS284643 | 5565.65 | 40194623 | 8038924600 | galu_Illumina_RNAseq_11C09096 | TRANSCRIPTOMIC | RANDOM |
| SRX118449 | Analysis of the Ganoderma lucidum transcriptome | Illumina HiSeq 2000 | Wuhan university | SRP006712 | The transcriptome sequencing for Ganoderma lucidum | SRS192681 | 349.42 | 3208334 | 577500120 | Ganoderma lucidum.fruiting.body | TRANSCRIPTOMIC | cDNA |
| SRX118690 | Analysis of the Ganoderma lucidum transcriptome | Illumina HiSeq 2000 | Wuhan university | SRP006712 | The transcriptome sequencing for Ganoderma lucidum | SRS192681 | 349.65 | 3219845 | 579572100 | Ganoderma lucidum.Slope.species | TRANSCRIPTOMIC | cDNA |
| SRX13785911 | Illumina sequencing of transcriptome, Ganoderma lucidum CGMCC 5.616, 2 days, biological replicate 1 | Illumina NovaSeq 6000 | Tianjin Institute of Industrial Biotechnology | SRP354811 | whole genome and transcriptome sequencing of Ganoderma lucidum CGMCC 5.616 | SRS11668593 | 1882.68 | 21844304 | 6553291200 | 2d_1 | TRANSCRIPTOMIC | RANDOM |
| SRX13785912 | Illumina sequencing of transcriptome, Ganoderma lucidum CGMCC 5.616, 2 days, biological replicate 2 | Illumina NovaSeq 6000 | Tianjin Institute of Industrial Biotechnology | SRP354811 | whole genome and transcriptome sequencing of Ganoderma lucidum CGMCC 5.616 | SRS11668594 | 1953.87 | 22710739 | 6813221700 | 2d_2 | TRANSCRIPTOMIC | RANDOM |
| SRX13785913 | Illumina sequencing of transcriptome, Ganoderma lucidum CGMCC 5.616,2 days, biological replicate 3 | Illumina NovaSeq 6000 | Tianjin Institute of Industrial Biotechnology | SRP354811 | whole genome and transcriptome sequencing of Ganoderma lucidum CGMCC 5.616 | SRS11668595 | 2020.05 | 23431888 | 7029566400 | 2d_3 | TRANSCRIPTOMIC | RANDOM |
| SRX13785914 | Illumina sequencing of transcriptome, Ganoderma lucidum CGMCC 5.616, 8 days, biological replicate 1 | Illumina NovaSeq 6000 | Tianjin Institute of Industrial Biotechnology | SRP354811 | whole genome and transcriptome sequencing of Ganoderma lucidum CGMCC 5.616 | SRS11668596 | 2055.1 | 23877597 | 7163279100 | 8d_1 | TRANSCRIPTOMIC | RANDOM |
| SRX13785915 | Illumina sequencing of transcriptome, Ganoderma lucidum CGMCC 5.616, 8 days, biological replicate 1 | Illumina NovaSeq 6000 | Tianjin Institute of Industrial Biotechnology | SRP354811 | whole genome and transcriptome sequencing of Ganoderma lucidum CGMCC 5.616 | SRS11668597 | 2254.88 | 25043434 | 7513030200 | 8d_2 | TRANSCRIPTOMIC | RANDOM |
| SRX13785916 | Illumina sequencing of transcriptome, Ganoderma lucidum CGMCC 5.616, 8 days, biological replicate 1 | Illumina NovaSeq 6000 | Tianjin Institute of Industrial Biotechnology | SRP354811 | whole genome and transcriptome sequencing of Ganoderma lucidum CGMCC 5.616 | SRS11668598 | 2416.9 | 27177162 | 8153148600 | 8d_3 | TRANSCRIPTOMIC | RANDOM |
| SRX13785917 | Illumina sequencing of transcriptome, Ganoderma lucidum CGMCC 5.616, 12 days, biological replicate 1 | Illumina NovaSeq 6000 | Tianjin Institute of Industrial Biotechnology | SRP354811 | whole genome and transcriptome sequencing of Ganoderma lucidum CGMCC 5.616 | SRS11668599 | 1951.55 | 22724825 | 6817447500 | 12d_1 | TRANSCRIPTOMIC | RANDOM |
| SRX13785918 | Illumina sequencing of transcriptome, Ganoderma lucidum CGMCC 5.616, 12 days, biological replicate 1 | Illumina NovaSeq 6000 | Tianjin Institute of Industrial Biotechnology | SRP354811 | whole genome and transcriptome sequencing of Ganoderma lucidum CGMCC 5.616 | SRS11668601 | 2111.44 | 24463322 | 7338996600 | 12d_2 | TRANSCRIPTOMIC | RANDOM |
| SRX13785919 | Illumina sequencing of transcriptome, Ganoderma lucidum CGMCC 5.616, 12 days, biological replicate 1 | Illumina NovaSeq 6000 | Tianjin Institute of Industrial Biotechnology | SRP354811 | whole genome and transcriptome sequencing of Ganoderma lucidum CGMCC 5.616 | SRS11668602 | 2002.19 | 23292862 | 6987858600 | 12d_3 | TRANSCRIPTOMIC | RANDOM |
| SRX14722902 | transcriptome of Ganoderma lucidum mycelium replicate 1 | Illumina NovaSeq 6000 | Institute of Botany, Jiangsu Province and Chinese | SRP367443 | transcriptome analysis of G. lucidum at five developmental stages | SRS12484998 | 2106.01 | 23718505 | 7115551500 | G1-1 | TRANSCRIPTOMIC | other |
| SRX14722903 | transcriptome of Ganoderma lucidum mycelium replicate 2 | Illumina NovaSeq 6000 | Institute of Botany, Jiangsu Province and Chinese | SRP367443 | transcriptome analysis of G. lucidum at five developmental stages | SRS12484997 | 2007.82 | 22237678 | 6671303400 | G1-2 | TRANSCRIPTOMIC | other |
| SRX14722904 | transcriptome of Ganoderma lucidum mature fruiting body replicate 2 | Illumina NovaSeq 6000 | Institute of Botany, Jiangsu Province and Chinese | SRP367443 | transcriptome analysis of G. lucidum at five developmental stages | SRS12484999 | 2671.21 | 29532041 | 8859612300 | G4-2 | TRANSCRIPTOMIC | other |
| SRX14722905 | transcriptome of Ganoderma lucidum mature fruiting body replicate 3 | Illumina NovaSeq 6000 | Institute of Botany, Jiangsu Province and Chinese | SRP367443 | transcriptome analysis of G. lucidum at five developmental stages | SRS12485000 | 2306.47 | 25603208 | 7680962400 | G4-3 | TRANSCRIPTOMIC | other |
| SRX14722906 | transcriptome of Ganoderma lucidum fruiting body in post-sporulation stage replicate 1 | Illumina NovaSeq 6000 | Institute of Botany, Jiangsu Province and Chinese | SRP367443 | transcriptome analysis of G. lucidum at five developmental stages | SRS12485001 | 2318.38 | 25627192 | 7688157600 | G5-1 | TRANSCRIPTOMIC | other |
| SRX14722907 | transcriptome of Ganoderma lucidum fruiting body in post-sporulation stage replicate 2 | Illumina NovaSeq 6000 | Institute of Botany, Jiangsu Province and Chinese | SRP367443 | transcriptome analysis of G. lucidum at five developmental stages | SRS12485003 | 2508.5 | 27834407 | 8350322100 | G5-2 | TRANSCRIPTOMIC | other |
| SRX14722908 | transcriptome of Ganoderma lucidum fruiting body in post-sporulation stage replicate 3 | Illumina NovaSeq 6000 | Institute of Botany, Jiangsu Province and Chinese | SRP367443 | transcriptome analysis of G. lucidum at five developmental stages | SRS12485002 | 2256.33 | 25054365 | 7516309500 | G5-3 | TRANSCRIPTOMIC | other |
| SRX14722909 | transcriptome of Ganoderma lucidum mycelium replicate 3 | Illumina NovaSeq 6000 | Institute of Botany, Jiangsu Province and Chinese | SRP367443 | transcriptome analysis of G. lucidum at five developmental stages | SRS12485004 | 2243.84 | 25028002 | 7508400600 | G1-3 | TRANSCRIPTOMIC | other |
| SRX14722910 | transcriptome of Ganoderma lucidum primordium replicate 1 | Illumina NovaSeq 6000 | Institute of Botany, Jiangsu Province and Chinese | SRP367443 | transcriptome analysis of G. lucidum at five developmental stages | SRS12485005 | 2302 | 25370776 | 7611232800 | G2-1 | TRANSCRIPTOMIC | other |
| SRX14722911 | transcriptome of Ganoderma lucidum primordium replicate 2 | Illumina NovaSeq 6000 | Institute of Botany, Jiangsu Province and Chinese | SRP367443 | transcriptome analysis of G. lucidum at five developmental stages | SRS12485006 | 2452.74 | 26719368 | 8015810400 | G2-2 | TRANSCRIPTOMIC | other |
| SRX14722912 | transcriptome of Ganoderma lucidum primordium replicate 3 | Illumina NovaSeq 6000 | Institute of Botany, Jiangsu Province and Chinese | SRP367443 | transcriptome analysis of G. lucidum at five developmental stages | SRS12485008 | 2090.51 | 23130677 | 6939203100 | G2-3 | TRANSCRIPTOMIC | other |
| SRX14722913 | transcriptome of Ganoderma lucidum young fruiting body replicate 1 | Illumina NovaSeq 6000 | Institute of Botany, Jiangsu Province and Chinese | SRP367443 | transcriptome analysis of G. lucidum at five developmental stages | SRS12485007 | 2397.32 | 26612145 | 7983643500 | G3-1 | TRANSCRIPTOMIC | other |
| SRX14722914 | transcriptome of Ganoderma lucidum young fruiting body replicate 2 | Illumina NovaSeq 6000 | Institute of Botany, Jiangsu Province and Chinese | SRP367443 | transcriptome analysis of G. lucidum at five developmental stages | SRS12485009 | 2029.65 | 22458749 | 6737624700 | G3-2 | TRANSCRIPTOMIC | other |
| SRX14722915 | transcriptome of Ganoderma lucidum young fruiting body replicate 3 | Illumina NovaSeq 6000 | Institute of Botany, Jiangsu Province and Chinese | SRP367443 | transcriptome analysis of G. lucidum at five developmental stages | SRS12485011 | 625.84 | 7178885 | 2153665500 | G3-3 | TRANSCRIPTOMIC | other |
| SRX14722916 | transcriptome of Ganoderma lucidum mature fruiting body replicate 1 | Illumina NovaSeq 6000 | Institute of Botany, Jiangsu Province and Chinese | SRP367443 | transcriptome analysis of G. lucidum at five developmental stages | SRS12485010 | 2453.21 | 27076287 | 8122886100 | G4-1 | TRANSCRIPTOMIC | other |
| SRX17149801 | Control_1 | Illumina HiSeq 4000 | Fujian Agriculture And Forestry University | SRP393031 | Transcriptome of Ganoderma lucidum | SRS14721835 | 2407.37 | 22405593 | 6721677900 | GL1 | TRANSCRIPTOMIC | cDNA |
| SRX17149802 | Control_2 | Illumina HiSeq 4000 | Fujian Agriculture And Forestry University | SRP393031 | Transcriptome of Ganoderma lucidum | SRS14721836 | 2417.05 | 22445691 | 6733707300 | GL2 | TRANSCRIPTOMIC | cDNA |
| SRX17149803 | Control_3 | Illumina HiSeq 4000 | Fujian Agriculture And Forestry University | SRP393031 | Transcriptome of Ganoderma lucidum | SRS14721837 | 2397.24 | 22437521 | 6731256300 | GL3 | TRANSCRIPTOMIC | cDNA |
| SRX17149804 | 200 M MeJA_1 | Illumina HiSeq 4000 | Fujian Agriculture And Forestry University | SRP393031 | Transcriptome of Ganoderma lucidum | SRS14721838 | 2531.9 | 22228643 | 6668592900 | GL4 | TRANSCRIPTOMIC | cDNA |
| SRX17149805 | 200 M MeJA_2 | Illumina HiSeq 4000 | Fujian Agriculture And Forestry University | SRP393031 | Transcriptome of Ganoderma lucidum | SRS14721839 | 2538.29 | 22179767 | 6653930100 | GL5 | TRANSCRIPTOMIC | cDNA |
| SRX17149806 | 200 M MeJA_3 | Illumina HiSeq 4000 | Fujian Agriculture And Forestry University | SRP393031 | Transcriptome of Ganoderma lucidum | SRS14721840 | 2554.08 | 22317134 | 6695140200 | GL6 | TRANSCRIPTOMIC | cDNA |
| SRX17149807 | 300 M MeJA_1 | Illumina HiSeq 4000 | Fujian Agriculture And Forestry University | SRP393031 | Transcriptome of Ganoderma lucidum | SRS14721841 | 2563.32 | 22374975 | 6712492500 | GL7 | TRANSCRIPTOMIC | cDNA |
| SRX17149808 | 300 M MeJA_2 | Illumina HiSeq 4000 | Fujian Agriculture And Forestry University | SRP393031 | Transcriptome of Ganoderma lucidum | SRS14721842 | 2498.87 | 21732606 | 6519781800 | GL8 | TRANSCRIPTOMIC | cDNA |
| SRX17149809 | 300 M MeJA_3 | Illumina HiSeq 4000 | Fujian Agriculture And Forestry University | SRP393031 | Transcriptome of Ganoderma lucidum | SRS14721843 | 2549.48 | 22328436 | 6698530800 | GL9 | TRANSCRIPTOMIC | cDNA |
| SRX2566119 | GSM2491764: Mycelia rep1; Ganoderma lucidum G.260125-1; ssRNA-seq | Illumina HiSeq 2500 | GEO | SRP100028 | Genome-wide Identification and Characterization of Natural Antisense Transcripts by Strand-specific RNA Sequencing in Ganoderma lucidum | SRS1982530 | 3269.04 | 25297045 | 5059409000 |  | TRANSCRIPTOMIC | cDNA |
| SRX2566120 | GSM2491765: Primordia rep1; Ganoderma lucidum G.260125-1; ssRNA-seq | Illumina HiSeq 2500 | GEO | SRP100028 | Genome-wide Identification and Characterization of Natural Antisense Transcripts by Strand-specific RNA Sequencing in Ganoderma lucidum | SRS1982531 | 3117.07 | 24160829 | 4832165800 |  | TRANSCRIPTOMIC | cDNA |
| SRX2566122 | GSM2491766: Fruiting bodies rep1; Ganoderma lucidum G.260125-1; ssRNA-seq | Illumina HiSeq 2500 | GEO | SRP100028 | Genome-wide Identification and Characterization of Natural Antisense Transcripts by Strand-specific RNA Sequencing in Ganoderma lucidum | SRS1982533 | 3656.61 | 28274255 | 5654851000 |  | TRANSCRIPTOMIC | cDNA |
| SRX2739542 | transcriptome data of GL | Illumina HiSeq 2000 | Sun yat-sen University | SRP102883 | transcriptome responses of Ganoderma lucidum to nitric oxide | SRS2093675 | 1555.12 | 15625225 | 3906306250 |  | TRANSCRIPTOMIC | size fractionation |
| SRX2739544 | transcriptome data of GL | Illumina HiSeq 2000 | Sun yat-sen University | SRP102883 | transcriptome responses of Ganoderma lucidum to nitric oxide | SRS2092462 | 1475.24 | 14766350 | 3691587500 |  | TRANSCRIPTOMIC | size fractionation |
| SRX2835005 | RNA-Seq of Ganoderma lucidum | Illumina HiSeq 2000 | Soil and Fertilizer Institute | SRP107384 | transcriptome data of Ganoderma lucidum strain yuanzhi8 | SRS2209345 | 1398.01 | 13772719 | 4131815700 | R201605014 | TRANSCRIPTOMIC | RANDOM |
| SRX2835006 | RNA-Seq of Ganoderma lucidum | Illumina HiSeq 2000 | Soil and Fertilizer Institute | SRP107384 | transcriptome data of Ganoderma lucidum strain yuanzhi8 | SRS2209345 | 1523.21 | 14847490 | 4454247000 | R201605020 | TRANSCRIPTOMIC | RANDOM |
| SRX2835007 | RNA-Seq of Ganoderma lucidum | Illumina HiSeq 2000 | Soil and Fertilizer Institute | SRP107384 | transcriptome data of Ganoderma lucidum strain yuanzhi8 | SRS2209345 | 1856.9 | 16551948 | 4965584400 | R201605021 | TRANSCRIPTOMIC | RANDOM |
| SRX2835008 | RNA-Seq of Ganoderma lucidum | Illumina HiSeq 2000 | Soil and Fertilizer Institute | SRP107384 | transcriptome data of Ganoderma lucidum strain yuanzhi8 | SRS2209345 | 1640.48 | 14570976 | 4371292800 | R201605015 | TRANSCRIPTOMIC | RANDOM |
| SRX2835009 | RNA-Seq of Ganoderma lucidum | Illumina HiSeq 2000 | Soil and Fertilizer Institute | SRP107384 | transcriptome data of Ganoderma lucidum strain yuanzhi8 | SRS2209345 | 1865.14 | 16679058 | 5003717400 | R201605022 | TRANSCRIPTOMIC | RANDOM |
| SRX2835010 | RNA-Seq of Ganoderma lucidum | Illumina HiSeq 2000 | Soil and Fertilizer Institute | SRP107384 | transcriptome data of Ganoderma lucidum strain yuanzhi8 | SRS2209345 | 1745.01 | 15570994 | 4671298200 | R201605023 | TRANSCRIPTOMIC | RANDOM |
| SRX2835011 | RNA-Seq of Ganoderma lucidum | Illumina HiSeq 2000 | Soil and Fertilizer Institute | SRP107384 | transcriptome data of Ganoderma lucidum strain yuanzhi8 | SRS2209345 | 1656.7 | 16272787 | 4881836100 | R201605012 | TRANSCRIPTOMIC | RANDOM |
| SRX2835012 | RNA-Seq of Ganoderma lucidum | Illumina HiSeq 2000 | Soil and Fertilizer Institute | SRP107384 | transcriptome data of Ganoderma lucidum strain yuanzhi8 | SRS2209345 | 1527.57 | 15094124 | 4528237200 | R201605013 | TRANSCRIPTOMIC | RANDOM |
| SRX2835013 | RNA-Seq of Ganoderma lucidum | Illumina HiSeq 2000 | Soil and Fertilizer Institute | SRP107384 | transcriptome data of Ganoderma lucidum strain yuanzhi8 | SRS2209345 | 1718.84 | 16854990 | 5056497000 | R201605018 | TRANSCRIPTOMIC | RANDOM |
| SRX2835014 | RNA-Seq of Ganoderma lucidum | Illumina HiSeq 2000 | Soil and Fertilizer Institute | SRP107384 | transcriptome data of Ganoderma lucidum strain yuanzhi8 | SRS2209345 | 1468.28 | 14219978 | 4265993400 | R201605019 | TRANSCRIPTOMIC | RANDOM |
| SRX2835015 | RNA-Seq of Ganoderma lucidum | Illumina HiSeq 2000 | Soil and Fertilizer Institute | SRP107384 | transcriptome data of Ganoderma lucidum strain yuanzhi8 | SRS2209345 | 1697.56 | 15180283 | 4554084900 | R201605016 | TRANSCRIPTOMIC | RANDOM |
| SRX2835016 | RNA-Seq of Ganoderma lucidum | Illumina HiSeq 2000 | Soil and Fertilizer Institute | SRP107384 | transcriptome data of Ganoderma lucidum strain yuanzhi8 | SRS2209345 | 1863.5 | 16643429 | 4993028700 | R201605017 | TRANSCRIPTOMIC | RANDOM |
| SRX3177401 | RNA-Seq of Ganoderma lucidum: compost mycelium | Illumina HiSeq 3000 | Shanghai Academy of Agriculture Sciences | SRP117291 | transcirptomic sequencing of compost mycelium from Ganoderma lucidum G0119 | SRS2506997 | 1868.11 | 36159403 | 5423910450 | Phase 1 replicate 1 | TRANSCRIPTOMIC | cDNA |
| SRX3177717 | RNA-Seq of Ganoderma lucidum: compost mycelium | Illumina HiSeq 3000 | Shanghai Academy of Agriculture Sciences | SRP117291 | transcirptomic sequencing of compost mycelium from Ganoderma lucidum G0119 | SRS2506997 | 2055.55 | 36159403 | 5423910450 | Phase 1 replicate 2 | TRANSCRIPTOMIC | cDNA |
| SRX3177719 | RNA-Seq of Ganoderma lucidum: compost mycelium | Illumina HiSeq 3000 | Shanghai Academy of Agriculture Sciences | SRP117291 | transcirptomic sequencing of compost mycelium from Ganoderma lucidum G0119 | SRS2506997 | 1265.19 | 24099061 | 3614859150 | Phase 2 replicate 1 | TRANSCRIPTOMIC | cDNA |
| SRX3177815 | RNA-Seq of Ganoderma lucidum: compost mycelium | Illumina HiSeq 3000 | Shanghai Academy of Agriculture Sciences | SRP117291 | transcirptomic sequencing of compost mycelium from Ganoderma lucidum G0119 | SRS2506997 | 1402.35 | 24099061 | 3614859150 | Phase 2 replicate 2 | TRANSCRIPTOMIC | cDNA |
| SRX3185766 | RNA-Seq of Ganoderma lucidum: compost mycelium | Illumina HiSeq 3000 | Shanghai Academy of Agriculture Sciences | SRP117291 | transcirptomic sequencing of compost mycelium from Ganoderma lucidum G0119 | SRS2506997 | 1857.31 | 36270697 | 5440604550 | Phase 3 replicate 1 | TRANSCRIPTOMIC | cDNA |
| SRX3185800 | RNA-Seq of Ganoderma lucidum: compost mycelium | Illumina HiSeq 3000 | Shanghai Academy of Agriculture Sciences | SRP117291 | transcirptomic sequencing of compost mycelium from Ganoderma lucidum G0119 | SRS2506997 | 2086.52 | 36270697 | 5440604550 | Phase 3 replicate 2 | TRANSCRIPTOMIC | cDNA |
| SRX3185920 | RNA-Seq of Ganoderma lucidum: compost mycelium | Illumina HiSeq 3000 | Shanghai Academy of Agriculture Sciences | SRP117291 | transcirptomic sequencing of compost mycelium from Ganoderma lucidum G0119 | SRS2506997 | 1648.17 | 32102746 | 4815411900 | Phase 4 replicate 1 | TRANSCRIPTOMIC | cDNA |
| SRX3188232 | RNA-Seq of Ganoderma lucidum: compost mycelium | Illumina HiSeq 3000 | Shanghai Academy of Agriculture Sciences | SRP117291 | transcirptomic sequencing of compost mycelium from Ganoderma lucidum G0119 | SRS2506997 | 1873.67 | 32102746 | 4815411900 | Phase 4 replicate 2 | TRANSCRIPTOMIC | cDNA |
| SRX3188342 | RNA-Seq of Ganoderma lucidum: compost mycelium | Illumina HiSeq 3000 | Shanghai Academy of Agriculture Sciences | SRP117291 | transcirptomic sequencing of compost mycelium from Ganoderma lucidum G0119 | SRS2506997 | 2008.96 | 38864755 | 5829713250 | Phase 5 replicate 1 | TRANSCRIPTOMIC | cDNA |
| SRX3191008 | RNA-Seq of Ganoderma lucidum: compost mycelium | Illumina HiSeq 3000 | Shanghai Academy of Agriculture Sciences | SRP117291 | transcirptomic sequencing of compost mycelium from Ganoderma lucidum G0119 | SRS2518385 | 2237.66 | 38864755 | 5829713250 | Phase 5 replicate 2 | TRANSCRIPTOMIC | cDNA |
| SRX3410445 | Effects of heat stress on Ganoderma lucidum | Illumina HiSeq 2000 | China Agricultural University | SRP125332 | Ganoderma lucidum Transcriptome or Gene expression | SRS2703684 | 10557.24 | 87601253 | 26280375900 |  | TRANSCRIPTOMIC | cDNA |
| SRX3410446 | Effects of heat stress on Ganoderma lucidum | Illumina HiSeq 2000 | China Agricultural University | SRP125332 | Ganoderma lucidum Transcriptome or Gene expression | SRS2703685 | 9338.94 | 77598425 | 23279527500 | HS | TRANSCRIPTOMIC | cDNA |
| SRX3507638 | RNA seq of Ganoderma Copper Induced | Illumina HiSeq 2500 | Mahrahshi Dayanand University, Rohtak | SRP127479 | Fungal Transcriptome | SRS2785164 | 850.98 | 13041686 | 2554009610 | Ganoderma Copper Induced | TRANSCRIPTOMIC | RT-PCR |
| SRX3507639 | RNA seq of Ganoderma Control | Illumina HiSeq 2500 | Mahrahshi Dayanand University, Rohtak | SRP127479 | Fungal Transcriptome | SRS2785164 | 1250.76 | 17856538 | 3389253868 | Ganoderma Control | TRANSCRIPTOMIC | RT-PCR |
| SRX4890249 | GSM3430624: WT rep1; Ganoderma lucidum; RNA-Seq | HiSeq X Ten | GEO | SRP165922 | Hydrogen sulfide, a novel small molecule signalling agent, participates in GAs biosynthesis under heat stress and the regulation of multiple signaling pathway genes in Ganoderma lucidum | SRS3937724 | 2752.09 | 28409593 | 8522877900 |  | TRANSCRIPTOMIC | cDNA |
| SRX4890250 | GSM3430625: WT rep2; Ganoderma lucidum; RNA-Seq | HiSeq X Ten | GEO | SRP165922 | Hydrogen sulfide, a novel small molecule signalling agent, participates in GAs biosynthesis under heat stress and the regulation of multiple signaling pathway genes in Ganoderma lucidum | SRS3937725 | 2131.05 | 22709280 | 6812784000 |  | TRANSCRIPTOMIC | cDNA |
| SRX4890251 | GSM3430626: WT rep3; Ganoderma lucidum; RNA-Seq | HiSeq X Ten | GEO | SRP165922 | Hydrogen sulfide, a novel small molecule signalling agent, participates in GAs biosynthesis under heat stress and the regulation of multiple signaling pathway genes in Ganoderma lucidum | SRS3937726 | 2134.05 | 22859464 | 6857839200 |  | TRANSCRIPTOMIC | cDNA |
| SRX4890252 | GSM3430627: WT+HS rep1; Ganoderma lucidum; RNA-Seq | HiSeq X Ten | GEO | SRP165922 | Hydrogen sulfide, a novel small molecule signalling agent, participates in GAs biosynthesis under heat stress and the regulation of multiple signaling pathway genes in Ganoderma lucidum | SRS3937727 | 1750.8 | 19206984 | 5762095200 |  | TRANSCRIPTOMIC | cDNA |
| SRX4890253 | GSM3430628: WT+HS rep2; Ganoderma lucidum; RNA-Seq | HiSeq X Ten | GEO | SRP165922 | Hydrogen sulfide, a novel small molecule signalling agent, participates in GAs biosynthesis under heat stress and the regulation of multiple signaling pathway genes in Ganoderma lucidum | SRS3937728 | 2293.69 | 24107789 | 7232336700 |  | TRANSCRIPTOMIC | cDNA |
| SRX4890254 | GSM3430629: WT+HS rep3; Ganoderma lucidum; RNA-Seq | HiSeq X Ten | GEO | SRP165922 | Hydrogen sulfide, a novel small molecule signalling agent, participates in GAs biosynthesis under heat stress and the regulation of multiple signaling pathway genes in Ganoderma lucidum | SRS3937729 | 1695.5 | 18581350 | 5574405000 |  | TRANSCRIPTOMIC | cDNA |
| SRX4890255 | GSM3430630: WT+NaHS+HS rep1; Ganoderma lucidum; RNA-Seq | HiSeq X Ten | GEO | SRP165922 | Hydrogen sulfide, a novel small molecule signalling agent, participates in GAs biosynthesis under heat stress and the regulation of multiple signaling pathway genes in Ganoderma lucidum | SRS3937730 | 2123.95 | 22798629 | 6839588700 |  | TRANSCRIPTOMIC | cDNA |
| SRX4890256 | GSM3430631: WT+NaHS+HS rep2; Ganoderma lucidum; RNA-Seq | HiSeq X Ten | GEO | SRP165922 | Hydrogen sulfide, a novel small molecule signalling agent, participates in GAs biosynthesis under heat stress and the regulation of multiple signaling pathway genes in Ganoderma lucidum | SRS3937731 | 1780.19 | 19505238 | 5851571400 |  | TRANSCRIPTOMIC | cDNA |
| SRX4890257 | GSM3430632: WT+NaHS+HS rep3; Ganoderma lucidum; RNA-Seq | HiSeq X Ten | GEO | SRP165922 | Hydrogen sulfide, a novel small molecule signalling agent, participates in GAs biosynthesis under heat stress and the regulation of multiple signaling pathway genes in Ganoderma lucidum | SRS3937732 | 1808.41 | 19780283 | 5934084900 |  | TRANSCRIPTOMIC | cDNA |
| SRX4890258 | GSM3430633: Sicontrol rep1; Ganoderma lucidum; RNA-Seq | HiSeq X Ten | GEO | SRP165922 | Hydrogen sulfide, a novel small molecule signalling agent, participates in GAs biosynthesis under heat stress and the regulation of multiple signaling pathway genes in Ganoderma lucidum | SRS3937733 | 2489.46 | 26219352 | 7865805600 |  | TRANSCRIPTOMIC | cDNA |
| SRX4890259 | GSM3430634: Sicontrol rep2; Ganoderma lucidum; RNA-Seq | HiSeq X Ten | GEO | SRP165922 | Hydrogen sulfide, a novel small molecule signalling agent, participates in GAs biosynthesis under heat stress and the regulation of multiple signaling pathway genes in Ganoderma lucidum | SRS3937737 | 2208.24 | 23605588 | 7081676400 |  | TRANSCRIPTOMIC | cDNA |
| SRX4890260 | GSM3430635: Sicontrol rep3; Ganoderma lucidum; RNA-Seq | HiSeq X Ten | GEO | SRP165922 | Hydrogen sulfide, a novel small molecule signalling agent, participates in GAs biosynthesis under heat stress and the regulation of multiple signaling pathway genes in Ganoderma lucidum | SRS3937735 | 2343.18 | 24665235 | 7399570500 |  | TRANSCRIPTOMIC | cDNA |
| SRX4890261 | GSM3430636: Sicontrol+HS rep1; Ganoderma lucidum; RNA-Seq | HiSeq X Ten | GEO | SRP165922 | Hydrogen sulfide, a novel small molecule signalling agent, participates in GAs biosynthesis under heat stress and the regulation of multiple signaling pathway genes in Ganoderma lucidum | SRS3937736 | 2532.49 | 26400289 | 7920086700 |  | TRANSCRIPTOMIC | cDNA |
| SRX4890262 | GSM3430637: Sicontrol+HS rep2; Ganoderma lucidum; RNA-Seq | HiSeq X Ten | GEO | SRP165922 | Hydrogen sulfide, a novel small molecule signalling agent, participates in GAs biosynthesis under heat stress and the regulation of multiple signaling pathway genes in Ganoderma lucidum | SRS3937738 | 2258.07 | 24104087 | 7231226100 |  | TRANSCRIPTOMIC | cDNA |
| SRX4890263 | GSM3430638: Sicontrol+HS rep3; Ganoderma lucidum; RNA-Seq | HiSeq X Ten | GEO | SRP165922 | Hydrogen sulfide, a novel small molecule signalling agent, participates in GAs biosynthesis under heat stress and the regulation of multiple signaling pathway genes in Ganoderma lucidum | SRS3937739 | 1764.63 | 19347889 | 5804366700 |  | TRANSCRIPTOMIC | cDNA |
| SRX4890264 | GSM3430639: Sicontrol+NaHS+HS rep1; Ganoderma lucidum; RNA-Seq | HiSeq X Ten | GEO | SRP165922 | Hydrogen sulfide, a novel small molecule signalling agent, participates in GAs biosynthesis under heat stress and the regulation of multiple signaling pathway genes in Ganoderma lucidum | SRS3937740 | 2003.15 | 21959950 | 6587985000 |  | TRANSCRIPTOMIC | cDNA |
| SRX4890265 | GSM3430640: Sicontrol+NaHS+HS rep2; Ganoderma lucidum; RNA-Seq | HiSeq X Ten | GEO | SRP165922 | Hydrogen sulfide, a novel small molecule signalling agent, participates in GAs biosynthesis under heat stress and the regulation of multiple signaling pathway genes in Ganoderma lucidum | SRS3937742 | 1676.34 | 18413822 | 5524146600 |  | TRANSCRIPTOMIC | cDNA |
| SRX4890266 | GSM3430641: Sicontrol+NaHS+HS rep3; Ganoderma lucidum; RNA-Seq | HiSeq X Ten | GEO | SRP165922 | Hydrogen sulfide, a novel small molecule signalling agent, participates in GAs biosynthesis under heat stress and the regulation of multiple signaling pathway genes in Ganoderma lucidum | SRS3937741 | 1697.86 | 18603242 | 5580972600 |  | TRANSCRIPTOMIC | cDNA |
| SRX4890267 | GSM3430642: CBSi rep1; Ganoderma lucidum; RNA-Seq | HiSeq X Ten | GEO | SRP165922 | Hydrogen sulfide, a novel small molecule signalling agent, participates in GAs biosynthesis under heat stress and the regulation of multiple signaling pathway genes in Ganoderma lucidum | SRS3937743 | 2346.53 | 25840126 | 7752037800 |  | TRANSCRIPTOMIC | cDNA |
| SRX4890268 | GSM3430643: CBSi rep2; Ganoderma lucidum; RNA-Seq | HiSeq X Ten | GEO | SRP165922 | Hydrogen sulfide, a novel small molecule signalling agent, participates in GAs biosynthesis under heat stress and the regulation of multiple signaling pathway genes in Ganoderma lucidum | SRS3937744 | 1944.93 | 21449205 | 6434761500 |  | TRANSCRIPTOMIC | cDNA |
| SRX4890269 | GSM3430644: CBSi rep3; Ganoderma lucidum; RNA-Seq | HiSeq X Ten | GEO | SRP165922 | Hydrogen sulfide, a novel small molecule signalling agent, participates in GAs biosynthesis under heat stress and the regulation of multiple signaling pathway genes in Ganoderma lucidum | SRS3937745 | 2029.45 | 21850524 | 6555157200 |  | TRANSCRIPTOMIC | cDNA |
| SRX4890270 | GSM3430645: CBSi+HS rep1; Ganoderma lucidum; RNA-Seq | HiSeq X Ten | GEO | SRP165922 | Hydrogen sulfide, a novel small molecule signalling agent, participates in GAs biosynthesis under heat stress and the regulation of multiple signaling pathway genes in Ganoderma lucidum | SRS3937746 | 1712.99 | 18876324 | 5662897200 |  | TRANSCRIPTOMIC | cDNA |
| SRX4890271 | GSM3430646: CBSi+HS rep2; Ganoderma lucidum; RNA-Seq | HiSeq X Ten | GEO | SRP165922 | Hydrogen sulfide, a novel small molecule signalling agent, participates in GAs biosynthesis under heat stress and the regulation of multiple signaling pathway genes in Ganoderma lucidum | SRS3937747 | 2092.38 | 22363082 | 6708924600 |  | TRANSCRIPTOMIC | cDNA |
| SRX4890272 | GSM3430647: CBSi+HS rep3; Ganoderma lucidum; RNA-Seq | HiSeq X Ten | GEO | SRP165922 | Hydrogen sulfide, a novel small molecule signalling agent, participates in GAs biosynthesis under heat stress and the regulation of multiple signaling pathway genes in Ganoderma lucidum | SRS3937748 | 2116.29 | 22595765 | 6778729500 |  | TRANSCRIPTOMIC | cDNA |
| SRX4890273 | GSM3430648: CBSi+NaHS+HS rep1; Ganoderma lucidum; RNA-Seq | HiSeq X Ten | GEO | SRP165922 | Hydrogen sulfide, a novel small molecule signalling agent, participates in GAs biosynthesis under heat stress and the regulation of multiple signaling pathway genes in Ganoderma lucidum | SRS3937749 | 2723.95 | 29139536 | 8741860800 |  | TRANSCRIPTOMIC | cDNA |
| SRX4890274 | GSM3430649: CBSi+NaHS+HS rep2; Ganoderma lucidum; RNA-Seq | HiSeq X Ten | GEO | SRP165922 | Hydrogen sulfide, a novel small molecule signalling agent, participates in GAs biosynthesis under heat stress and the regulation of multiple signaling pathway genes in Ganoderma lucidum | SRS3937750 | 2092.59 | 22531812 | 6759543600 |  | TRANSCRIPTOMIC | cDNA |
| SRX4890275 | GSM3430650: CBSi+NaHS+HS rep3; Ganoderma lucidum; RNA-Seq | HiSeq X Ten | GEO | SRP165922 | Hydrogen sulfide, a novel small molecule signalling agent, participates in GAs biosynthesis under heat stress and the regulation of multiple signaling pathway genes in Ganoderma lucidum | SRS3937751 | 1763.55 | 19419945 | 5825983500 |  | TRANSCRIPTOMIC | cDNA |
| SRX6919656 | Ganoderma lucidum Raw sequence reads | Illumina HiSeq 2000 | Jiangnan University | SRP223606 | Ganoderma lucidum Raw sequence reads | SRS5450539 | 1884.38 | 20291966 | 6087589800 | Ganoderma lucidumRNA-Seq LSSC-4 | METATRANSCRIPTOMIC | RANDOM |
| SRX6919657 | Ganoderma lucidum Raw sequence reads | Illumina HiSeq 2000 | Jiangnan University | SRP223606 | Ganoderma lucidum Raw sequence reads | SRS5450539 | 1560.98 | 16484637 | 4945391100 | Ganoderma lucidumRNA-Seq LSSC-5 | METATRANSCRIPTOMIC | RANDOM |
| SRX6919658 | Ganoderma lucidum Raw sequence reads | Illumina HiSeq 2000 | Jiangnan University | SRP223606 | Ganoderma lucidum Raw sequence reads | SRS5450539 | 1344.94 | 14403009 | 4320902700 | Ganoderma lucidumRNA-Seq LSSC-6 | METATRANSCRIPTOMIC | RANDOM |
| SRX6919659 | Ganoderma lucidum Raw sequence reads | Illumina HiSeq 2000 | Jiangnan University | SRP223606 | Ganoderma lucidum Raw sequence reads | SRS5450539 | 2001.26 | 21726000 | 6517800000 | Ganoderma lucidumRNA-Seq LSSC-7 | METATRANSCRIPTOMIC | RANDOM |
| SRX6919660 | Ganoderma lucidum Raw sequence reads | Illumina HiSeq 2000 | Jiangnan University | SRP223606 | Ganoderma lucidum Raw sequence reads | SRS5450539 | 1596.8 | 17051213 | 5115363900 | Ganoderma lucidumRNA-Seq LSSC-8 | METATRANSCRIPTOMIC | RANDOM |
| SRX6919661 | Ganoderma lucidum Raw sequence reads | Illumina HiSeq 2000 | Jiangnan University | SRP223606 | Ganoderma lucidum Raw sequence reads | SRS5450539 | 1760.14 | 19046546 | 5713963800 | Ganoderma lucidumRNA-Seq SC-4 | METATRANSCRIPTOMIC | RANDOM |
| SRX6919662 | Ganoderma lucidum Raw sequence reads | Illumina HiSeq 2000 | Jiangnan University | SRP223606 | Ganoderma lucidum Raw sequence reads | SRS5450539 | 1367.07 | 14958176 | 4487452800 | Ganoderma lucidumRNA-Seq SC-5 | METATRANSCRIPTOMIC | RANDOM |
| SRX6919663 | Ganoderma lucidum Raw sequence reads | Illumina HiSeq 2000 | Jiangnan University | SRP223606 | Ganoderma lucidum Raw sequence reads | SRS5450539 | 1715.92 | 18123625 | 5437087500 | Ganoderma lucidumRNA-Seq SC-6 | METATRANSCRIPTOMIC | RANDOM |
| SRX6919664 | Ganoderma lucidum Raw sequence reads | Illumina HiSeq 2000 | Jiangnan University | SRP223606 | Ganoderma lucidum Raw sequence reads | SRS5450539 | 1792.78 | 19498796 | 5849638800 | Ganoderma lucidumRNA-Seq SC-7 | METATRANSCRIPTOMIC | RANDOM |
| SRX6919665 | Ganoderma lucidum Raw sequence reads | Illumina HiSeq 2000 | Jiangnan University | SRP223606 | Ganoderma lucidum Raw sequence reads | SRS5450539 | 1735.88 | 18699435 | 5609830500 | Ganoderma lucidumRNA-Seq SC-8 | METATRANSCRIPTOMIC | RANDOM |
| SRX7990348 | CK-1 0.5h | Illumina HiSeq 2500 | Jiangsu University | SRP253940 | Ganoderma lucidum Transcriptome or Gene expression | SRS6373932 | 2184.98 | 26838924 | 7448020553 | 1 | TRANSCRIPTOMIC | PCR |
| SRX7990349 | CK-2 0.5h | Illumina HiSeq 2500 | Jiangsu University | SRP253940 | Ganoderma lucidum Transcriptome or Gene expression | SRS6373932 | 2091.75 | 25679516 | 7117239950 | 2 | TRANSCRIPTOMIC | PCR |
| SRX7990350 | 28K-2 3h | Illumina HiSeq 2500 | Jiangsu University | SRP253940 | Ganoderma lucidum Transcriptome or Gene expression | SRS6373932 | 2095.51 | 25089383 | 7133568353 | 11 | TRANSCRIPTOMIC | PCR |
| SRX7990351 | 28K-3 3h | Illumina HiSeq 2500 | Jiangsu University | SRP253940 | Ganoderma lucidum Transcriptome or Gene expression | SRS6373932 | 2077.23 | 24846024 | 7065515650 | 12 | TRANSCRIPTOMIC | PCR |
| SRX7990352 | CK-3 0.5h | Illumina HiSeq 2500 | Jiangsu University | SRP253940 | Ganoderma lucidum Transcriptome or Gene expression | SRS6373932 | 2240.83 | 27478464 | 7640460376 | 3 | TRANSCRIPTOMIC | PCR |
| SRX7990353 | 28K-1 0.5h | Illumina HiSeq 2500 | Jiangsu University | SRP253940 | Ganoderma lucidum Transcriptome or Gene expression | SRS6373932 | 1965.02 | 24003296 | 6696986542 | 4 | TRANSCRIPTOMIC | PCR |
| SRX7990354 | 28K-2 0.5h | Illumina HiSeq 2500 | Jiangsu University | SRP253940 | Ganoderma lucidum Transcriptome or Gene expression | SRS6373932 | 2164.71 | 26510932 | 7357337677 | 5 | TRANSCRIPTOMIC | PCR |
| SRX7990355 | 28K-3 0.5h | Illumina HiSeq 2500 | Jiangsu University | SRP253940 | Ganoderma lucidum Transcriptome or Gene expression | SRS6373932 | 2140.57 | 26246939 | 7300712346 | 6 | TRANSCRIPTOMIC | PCR |
| SRX7990356 | CK-1 3h | Illumina HiSeq 2500 | Jiangsu University | SRP253940 | Ganoderma lucidum Transcriptome or Gene expression | SRS6373932 | 2005.83 | 24485755 | 6834977805 | 7 | TRANSCRIPTOMIC | PCR |
| SRX7990357 | CK-2 3h | Illumina HiSeq 2500 | Jiangsu University | SRP253940 | Ganoderma lucidum Transcriptome or Gene expression | SRS6373932 | 2116.33 | 25880333 | 7227193977 | 8 | TRANSCRIPTOMIC | PCR |
| SRX7990358 | CK-3 3h | Illumina HiSeq 2500 | Jiangsu University | SRP253940 | Ganoderma lucidum Transcriptome or Gene expression | SRS6373932 | 2032.22 | 24663534 | 6885124543 | 9 | TRANSCRIPTOMIC | PCR |
| SRX7990359 | 28K-1 3h | Illumina HiSeq 2500 | Jiangsu University | SRP253940 | Ganoderma lucidum Transcriptome or Gene expression | SRS6373932 | 2063.04 | 24631430 | 6994916250 | 10 | TRANSCRIPTOMIC | PCR |
| SRX8667526 | Ganoderma lucidum Raw sequence reads | Illumina HiSeq 2000 | Jiangnan University | SRP270030 | Ganoderma lucidum Raw sequence reads | SRS6947802 | 5845.29 | 68701273 | 20610381900 | Ganoderma lucidumRNA-Seq SC-4 | METATRANSCRIPTOMIC | RANDOM |
| SRX8667527 | Ganoderma lucidum Raw sequence reads | Illumina HiSeq 2000 | Jiangnan University | SRP270030 | Ganoderma lucidum Raw sequence reads | SRS6947802 | 6074.17 | 70817491 | 21245247300 | Ganoderma lucidumRNA-Seq SC-5 | METATRANSCRIPTOMIC | RANDOM |
| SRX8667528 | Ganoderma lucidum Raw sequence reads | Illumina HiSeq 2000 | Jiangnan University | SRP270030 | Ganoderma lucidum Raw sequence reads | SRS6947802 | 6057.21 | 70782536 | 21234760800 | Ganoderma lucidumRNA-Seq SC-6 | METATRANSCRIPTOMIC | RANDOM |
| SRX8667529 | Ganoderma lucidum Raw sequence reads | Illumina HiSeq 2000 | Jiangnan University | SRP270030 | Ganoderma lucidum Raw sequence reads | SRS6947802 | 5902.95 | 67708956 | 20312686800 | Ganoderma lucidumRNA-Seq SC-7 | METATRANSCRIPTOMIC | RANDOM |
| SRX8667530 | Ganoderma lucidum Raw sequence reads | Illumina HiSeq 2000 | Jiangnan University | SRP270030 | Ganoderma lucidum Raw sequence reads | SRS6947802 | 5828.05 | 68043055 | 20412916500 | Ganoderma lucidumRNA-Seq SC-8 | METATRANSCRIPTOMIC | RANDOM |
| SRX8667531 | Ganoderma lucidum Raw sequence reads | Illumina HiSeq 2000 | Jiangnan University | SRP270030 | Ganoderma lucidum Raw sequence reads | SRS6947802 | 6249.9 | 73128066 | 21938419800 | Ganoderma lucidumRNA-Seq LSSC-4 | METATRANSCRIPTOMIC | RANDOM |
| SRX8667532 | Ganoderma lucidum Raw sequence reads | Illumina HiSeq 2000 | Jiangnan University | SRP270030 | Ganoderma lucidum Raw sequence reads | SRS6947802 | 5851.36 | 68406034 | 20521810200 | Ganoderma lucidumRNA-Seq LSSC-5 | METATRANSCRIPTOMIC | RANDOM |
| SRX8667533 | Ganoderma lucidum Raw sequence reads | Illumina HiSeq 2000 | Jiangnan University | SRP270030 | Ganoderma lucidum Raw sequence reads | SRS6947802 | 4135.18 | 48082594 | 14424778200 | Ganoderma lucidumRNA-Seq LSSC-6 | METATRANSCRIPTOMIC | RANDOM |
| SRX8667534 | Ganoderma lucidum Raw sequence reads | Illumina HiSeq 2000 | Jiangnan University | SRP270030 | Ganoderma lucidum Raw sequence reads | SRS6947802 | 6010.94 | 69857954 | 20957386200 | Ganoderma lucidumRNA-Seq LSSC-7 | METATRANSCRIPTOMIC | RANDOM |
| SRX8667535 | Ganoderma lucidum Raw sequence reads | Illumina HiSeq 2000 | Jiangnan University | SRP270030 | Ganoderma lucidum Raw sequence reads | SRS6947802 | 6115.87 | 69528272 | 20858481600 | Ganoderma lucidumRNA-Seq LSSC-8 | METATRANSCRIPTOMIC | RANDOM |

Table S6. Abbreviated list.

| Abbreviations | | |
| --- | --- | --- |
| No. | Abbrreviation | Full name |
| 1 | G. lucidum | Ganoderma lucidum |
| 2 | GA | Gannoderic acid |
| 3 | TLR | toll-like receptors |
| 4 | MyD88 | myeloiddifferentiationfactor88 |
| 5 | JAK | janus kinase |
| 6 | TRAF6 | TNF receptor associated factor 6 |
| 7 | MAPK | mitogen-activated protein kinase |
| 8 | NF-Κb | nuclear factor kappa-B |
| 9 | MKK | MAP kinase kinase |
| 10 | IKKβ | inhibitor kappa B kinaseβ |
| 11 | STAT | signal transducers and activators of transcription |
| 12 | PI3K | phosphatidylinositide 3-kinases |
| 13 | p38 | p38 mitogen-activated protein kinase |
| 14 | JNK | c-Jun N-terminal kinase |
| 15 | p50 | NF-κB1 |
| 16 | p65 | RelA |
| 17 | IκB | inhibitor of NF-κB |
| 18 | Akt | protein kinase B |
| 19 | AP-1 | activator protein-1 |
| 20 | MMP-9 | matrix metalloproteinase-9 |
| 21 | IL-1β | interleukin-1β |
| 22 | TNF-α | tumor necrosis factor-α |
| 23 | CD8 | cluster of differentiation 8 |
| 24 | IL-10 | interleukin 10 |
| 25 | IFN-γ | interferon γ |
| 26 | AACT | actyl-CoA C-acetyltransferase |
| 27 | HMGS | 3-Hydroxy-3-methylglutaryl coenthase A synthase |
| 28 | HMGR | 3-hydroxy-3-methyl glutaryl coenzyme A reductase |
| 29 | MK | mevalonate kinase |
| 30 | MPK | phosphomevalonate kinase |
| 31 | MVD | mevalonate pyrophosphate decarboxylase |
| 32 | IPP | isopentenyl diphosphate |
| 33 | IDI | diphosphateisomerase |
| 34 | DMAPP | dimethylallyl pyrophosphate |
| 35 | FPS | farnesyl pyrophosphate synthase |
| 36 | FPP | Farnesyl pyrophosphate |
| 37 | SQS | squalene synthase |
| 38 | SE | squalene epoxidase |
| 39 | LSS | lanosterol synthase |
| 40 | HLDOA | 3-hydroxy-lanosta-8,24-dien-26-oic |
| 41 | DHLDOA | 3,28-dihydroxy-lanosta-8,24-dien-26-oic |
| 42 | MCC | microcrystalline cellulose |
| 43 | MeJA | methyl jasmonate |
| 44 | sdhB | succinate dehydrogenase |
